# Novel Organosilicon Tetramers with Dialkyl-Substituted [1]Benzothieno[3,2-b]benzothiophene Moieties for Solution-Processible Organic Electronics

**DOI:** 10.3390/molecules30234639

**Published:** 2025-12-03

**Authors:** Irina O. Gudkova, Evgeniy A. Zaborin, Alexander I. Buzin, Artem V. Bakirov, Yaroslava O. Titova, Oleg V. Borshchev, Sergey N. Chvalun, Sergey A. Ponomarenko

**Affiliations:** 1 Enikolopov Institute of Synthetic Polymeric Materials of the Russian Academy of Sciences, Profsoyuznaya Str. 70, Moscow 117393, Russia; zaborin@ispm.ru (E.A.Z.); buzinai@ispm.ru (A.I.B.); bakirov.artem@gmail.com (A.V.B.); yaroslava.titova@ispm.ru (Y.O.T.); borshchev@ispm.ru (O.V.B.); s-chvalun@yandex.ru (S.N.C.); 2 Kurchatov Institute National Research Center, 1 Akademika Kurchatova Sq., Moscow 123182, Russia

**Keywords:** organic semiconductors, [1]benzothieno[3,2-b]benzothiophene, organosilicon tetramers, phase transitions, X-ray diffraction analysis, organic field-effect transistors

## Abstract

The synthesis, phase behavior and semiconductor properties of two novel organosilicon tetramers with dialkyl-substituted [1]benzothieno[3,2-b]benzothiophene (BTBT) moieties, **D4-Und-BTBT-Hex** and **D4-Hex-BTBT-Oct**, are described. The synthesis of these molecules was carried out by sequential modification of the BTBT core by carbonyl-containing functional alkyl substituents using the Friedel–Crafts reaction, followed by the reduction in the keto group. The target tetramers, **D4-Und-BTBT-Hex** and **D4-Hex-BTBT-Oct**, were obtained by the hydrosilylation reaction between tetraallylsilane and corresponding 1,1,3,3-tetramethyl-1-(ω-(7-alkyl[1]benzothieno[3,2-b]benzothiophen-2-yl)alkyl)disiloxanes. The chemical structure of the compounds obtained was confirmed by NMR ^1^H-, ^13^C- and ^29^Si-spectroscopy, gel permeation chromatography and elemental analysis. Their phase behavior was investigated by differential scanning calorimetry, polarization optical microscopy and X-ray diffraction analysis. It was found that **D4-Und-BTBT-Hex** shows higher crystallinity at room temperature as compared to **D4-Hex-BTBT-Oct**, while both molecules possess smectic ordering favorable for active layer formation in organic field-effect transistors (OFETs). The active layers were applied by spin-coating under conditions of a homogeneous thin layer formation with a low content of defects. The devices obtained from **D4-Und-BTBT-Hex** have demonstrated good semiconductor characteristics in OFETs with a hole mobility up to 3.5 × 10^−2^ cm^2^ V^−1^ s^−1^, a low threshold voltage and an on/off ratio up to 10^7^.

## 1. Introduction

In the last decade, there has been significant interest in the development of novel functional materials for various organic electronic devices [1,2,3,4,5]. Organic semiconductors (OSCs) are widely used in organic field-effect transistors (OFETs) [6], organic light-emitting transistors (OLETs) [7], organic electrochemical transistors (OECTs) [8] and the gas and liquid sensors based on them, namely, organic and hybrid photovoltaics [9]. The active components in such devices are based on low-molecular, oligomeric or polymeric organic semiconductors, where the density of states is generally determined by the disordered nature of the molecular solid rather than energy bands [10]. The emergence of a large number of stable organic semiconductors makes it possible to produce various semiconductor devices that can combine all the advantages of organic molecules and conducting systems. The examples of such devices include organic light-emitting diodes (OLEDs) [11], organic photovoltaic cells (OPVs) [12], OFETs [13], chemical and biological sensors, artificial synapses and computing devices [14]. OSCs have a number of advantages, namely the following: the ability to controllably change properties, internal flexibility, relatively low cost and a wide range of applications [15,16,17]. In turn, low-molecular weight and oligomeric OSCs have a number of advantages over the conjugated polymer molecules, such as a strictly defined molecular structure, the absence of defects and reproducibility from batch to batch [18,19]. In this case, oligomeric OSCs are understood as compounds that, unlike classical oligomers, have a strictly defined molecular structure, and, unlike low-molecular weight compounds, cannot be purified by sublimation even in a deep vacuum due to their sufficiently high molecular weight. Their physical and chemical properties can be easily and effectively controlled by modifying functional groups and designing suitable molecular structures. The group of [1]Benzothieno[3,2-b]benzothiophene (BTBT) derivatives is of great interest as low-molecular weight and oligomeric OSCs. Various BTBT derivatives are widely used in different devices (OFETs, OLEDs), due to their high conductivity, good solubility in organic solvents and relatively high stability in air [20]. The chemical properties of BTBT provide the possibility of introducing alkyl, aryl, alkenyl, alkynyl, alkoxyl, carboxyl and other substituents [21,22] into any position of the BTBT annulated core, as well as obtaining symmetrical and asymmetrical derivatives [23,24]. In turn, the functionalization of BTBT derivatives with organosilicon fragments provides flexibility to such compounds while maintaining the semiconductor properties of BTBT. It was previously shown that dimeric organosilicon structures containing alkyl spacers between tetramethyldisiloxane and BTBT units can be used as an active semiconductor layer in OFETs. Ultrathin-layer OFETs manufactured using the Langmuir–Blodgett or Langmuir–Schaeffer technique, based on such dimers, have demonstrated good charge carrier mobilities ranging from 10^−4^ to 10^−1^ cm^2^ V^−1^ s^−1^ [25,26,27,28].

**Figure 1:**
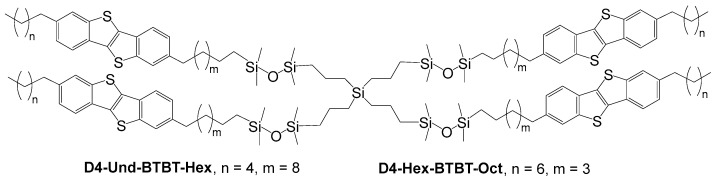
Chemical structures of flexible tetramers based on four dialkyl-substituted BTBT moieties, with flexible carbosilane–siloxane fragments.

In this work, we synthesized and investigated novel tetrameric structures containing four 2,7-dialkyl-substituted BTBT semiconductor moieties with different spacer and terminal alkyl chain lengths attached to a flexible carbosilane–siloxane core (Figure 1). Similar structures using quaterthiophene as a semiconductor moiety have demonstrated good semiconducting properties in OFETs [29] prepared by solution processing. The tetramers with longer oligothiophenes, containing from 5 to 7 conjugated 2,5-thienyl fragments, have shown interesting phase behavior and intramolecular aggregation in solutions [30].

## 2. Results

### 2.1. Synthesis

**Scheme 1:**
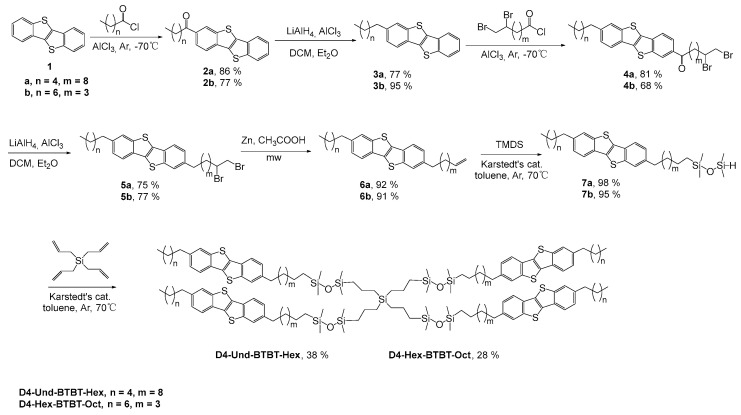
Synthesis of compounds **D4-Und-BTBT-Hex**, **D4-Hex-BTBT-Oct**.

In this work, the synthesis of *tetrakis*(1-(1,1,3,3-tetramethyl-3-(ω-(7-alkyl-[1]Benzothieno[3,2-b]benzothiophen-2-yl)alkyl)disiloxanyl)propyl)silanes **D4-Und-BTBT-Hex** and **D4-Hex-BTBT-Oct** was carried out in order to study their phase behavior and semiconductor properties. The synthesis of the target molecules was carried out by sequential acylation of BTBT according to Friedel–Crafts, followed by a reduction in the keto group [31]. The sequential introduction of alkyl substituents is due to the asymmetry of the molecule—one alkyl was unfunctional to serve as the terminal aliphatic group and the other alkyl contained a bromine-protected double bond as a functionality used for the following reactions. At the second stage, the reaction of debromination of bromine protection was carried out with the formation of a terminal double bond, followed by the addition of tetramethyldisiloxane (TMDS) [25]. At the final stage of the synthesis, the hydrosilylation reaction of the corresponding 1,1,3,3-tetramethyl-1-(6-(ω-alkyl-[1]Benzothieno[3,2-b]benzothiophen-2-yl)alkyl)disiloxanes **7a**, **b** with tetraallylsilane in a ratio of 1:5 in the presence of Karstedt’s (Karstedt’s catalyst: CAS 68478-92-2) catalyst was carried out (Scheme 1).

The reaction progress was monitored by gel-permission chromatography (GPC) and thin-layer chromatography (TLC).

**Figure 2:**
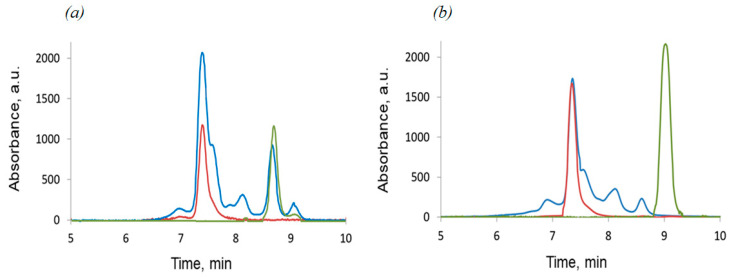
GPC chromatogram monitoring of the synthesis of (**a**) **D4-Und-BTBT-Hex**: starting compound **7a** (green), reaction mixture (blue), target compound (red); (**b**) **D4-Hex-BTBT-Oct**: starting compound **7b** (green), reaction mixture (blue), target compound (red) (GPC chromatograms are in Figures S24–S27, ESI).

Low yields of reaction products at the last stage are due to the formation of mono-, di- and tri-addition by-products, which is confirmed by GPC (Figure 2). However, the reagent ratio changing has not increased the formation of the target tetra-addition product. Purification of the target compound was carried out by column chromatography, using cyclohexane/toluene 10:1 vol/vol mixture as an eluent.

The compounds obtained were characterized by ^1^H, ^13^C and ^29^Si NMR spectroscopy, mass spectrometry MALDI and elemental analysis (see below in Section 3 as well as spectra Figures S1–S23, Figures S28 and S29, ESI). A distinctive feature of the ^1^H NMR spectra of both compounds, **D4-Und-BTBT-Hex** and **D4-Hex-BTBT-Oct**, is the absence of a signal from the terminal proton at silicon in the region of 4.67 ppm, as well as a doublet in the region of 0.15 ppm, corresponding to six protons of two methyl groups at silicon containing this proton.

### 2.2. Thermal Properties and Phase Behavior

**Figure 3:**
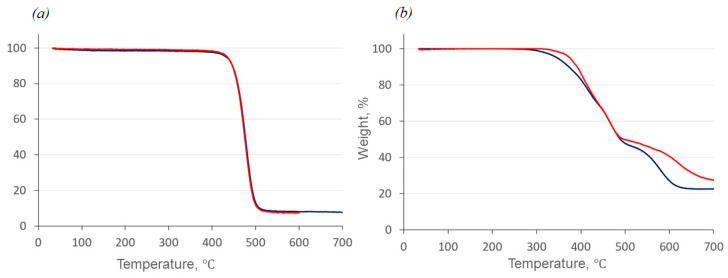
Results of thermogravimetric analysis of compounds **D4-Und-BTBT-Hex** (blue curves) and **D4-Hex-BTBT-Oct** (red curves): (**a**) in the argon atmosphere; (**b**) in the air atmosphere.

The thermal and thermo-oxidative stability of the compounds obtained, **D4-Und-BTBT-Hex** and **D4-Hex-BTBT-Oct**, were investigated by thermogravimetric analysis (TGA) in the argon atmosphere and in the air, respectively (Figure 3).

The results of thermogravimetric analysis have shown that the stabilities of both compounds, **D4-Und-BTBT-Hex** and **D4-Hex-BTBT-Oct**, are very close: the temperatures of 5% mass loss are 433–435 °C in the inert atmosphere and 346–375 °C in the air. The lower stability of compound **D4-Und-BTBT-Hex** in the air can be explained by the presence of longer alkyl substituents in the molecule. TGA performed in air shows a two-stage mass loss, with the first stage representing the loss of alkyl groups and the second stage representing the oxidation of sulfur in BTBT at higher temperature in oxygen [32].

The packing of molecules and their ability to self-organize have a significant effect on the semiconducting properties of the final material [33]. The phase behavior of **D4-Und-BTBT-Hex** and **D4-Hex-BTBT-Oct** samples in the bulk was studied by the differential scanning calorimetry (DSC) and polarization optical microscopy (POM) methods.

**Figure 4:**
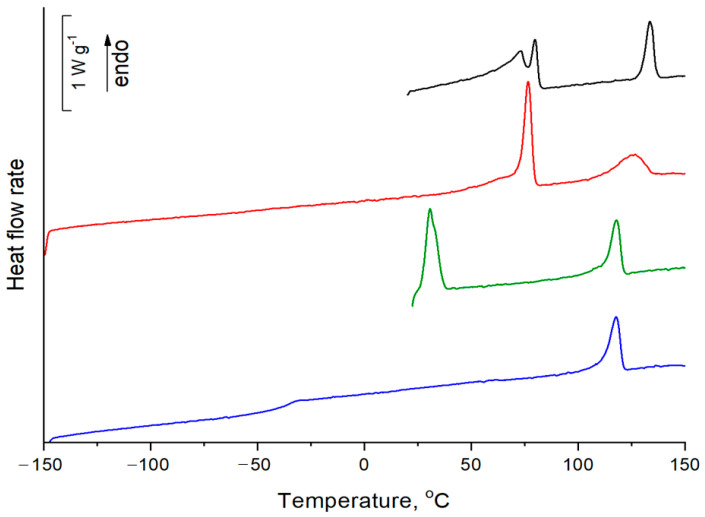
The DSC curves: **D4-Und-BTBT-Hex:** the 1st heating—black curve, the 2nd heating—red curve; **D4-Hex-BTBT-Oct:** the 1st heating—green curve, the 2nd heating—blue curve.

The DSC scans of the first and second heating of both compounds are shown in Figure 4. The heating of a virgin sample of **D4-Und-BTBT-Hex** is accompanied by two endothermic peaks. The first one is bimodal with maxima at 73 and 80 °C, while the second one is observed at 134 °C. On the second heating after slow cooling (20 °C min^−1^), two peaks are also observed at 77 and 127 °C. The bimodality of the first peak is reduced to a low temperature shoulder. The heating of a virgin sample of **D4-Hex-BTBT-Oct** reveals two endothermic peaks at 31 and 118 °C. However, a low temperature phase is not formed after melt crystallization. The second heating of **D4-Hex-BTBT-Oct** is accompanied by the jump in heat capacity at −40 °C (Δ*C*_p_ = 0.29 J g^−1^ K^−1^), corresponding to the glass transition of amorphous phase and an endothermic peak at 118 °C. It is assumed that the thermophysical effects at low temperatures correspond to the glass transition and crystallization of flexible alkyl groups [34], while the transitions in the range of 118–127 °C correspond to the disordering of rigid benzothiophene groups and the subsequent isotropization of the material. (Temperatures and enthalpies of transitions are summarized in Table S1, ESI.)

**Figure 5:**
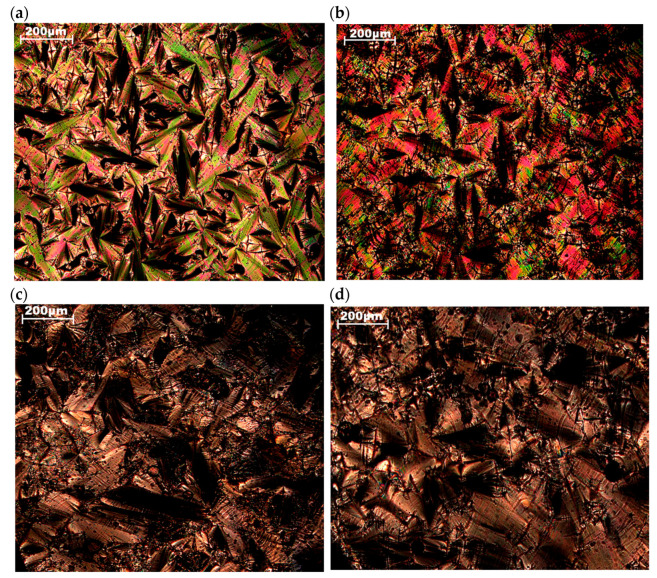
Micrographs in crossed polarizers during cooling: (**a**) sample **D4-Und-BTBT-Hex** at 110 °C, (**b**) sample **D4-Und-BTBT-Hex** at 40 °C, (**c**) sample **D4-Hex-BTBT-Oct** at 97 °C, (**d**) sample **D4-Hex-BTBT-Oct** at 5 °C.

To determine the nature of the high-temperature phases, samples **D4-Und-BTBT-Hex** and **D4-Hex-BTBT-Oct** were investigated by POM in crossed polarizers (Figure 5). According to the data obtained, compound **D4-Und-BTBT-Hex** is characterized by the presence of birefringence in the temperature range from room temperature (ca. 23 °C) until 120 °C, while there were observed texture changes near 70 °C, and further heating led to a transition to an isotropic melt. However, only a transition to an isotropic melt is observed for **D4-Hex-BTBT-Oct** at the temperature of 118 °C. Both compounds demonstrate the formation of a fan-shaped structure when cooling the samples from the isotropic melts (Figure 5a,c), which is typical for disordered smectic mesophases A or C. However, further cooling of **D4-Und-BTBT-Hex** below 70 °C is characterized by the braking of the fans, indicating some ordering process (Figure 5b), corresponding to the transition to a crystal or one of the ordered smectic phases (i.e., E, G, F or K). Further cooling the sample of **D4-Hex-BTBT-Oct** does not lead to any significant changes in the texture (Figure 5d).

### 2.3. X-Ray Diffraction

**Figure 6:**
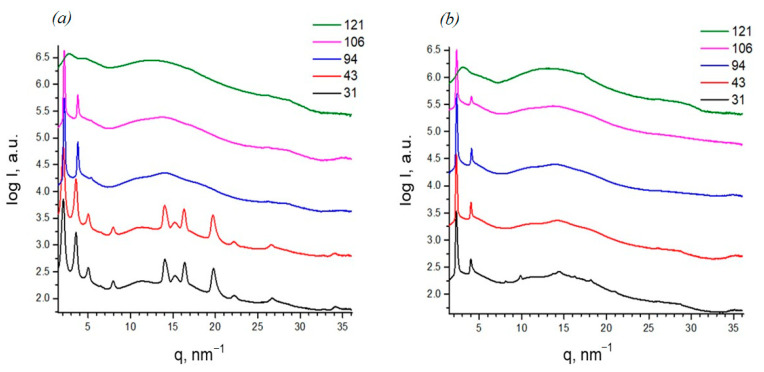
SWAXS diffraction patterns at elevated temperatures: (**a**) sample **D4-Und-BTBT-Hex**; (**b**) sample **D4-Hex-BTBT-Oct**. Curves are shown in logarithmic scale and shifted for clarity. (Diffraction patterns in Figure S30, ESI).

To determine the structure and phase behavior, small- and wide-angle X-ray diffraction analysis was performed during in situ heating (Figure 6). The diffraction pattern of sample **D4-Und-BTBT-Hex** at room temperature shows a set of reflections that clearly indicate the type of ordering as smectic E (SmE). Wide-angle reflections in the region of q 14–22 nm^−1^ refer to the orthorhombic lattice of BTBT fragments and are indexed as 110, 020 and 120. The position of the small-angle peak, as well as its orders, allows us to determine a layer period equal to 30.4 Å. The obtained value is less than 35.8 Å reported previously for the dimer of a similar structure with hexyl substituents **D2-Und-BTBT-Hex** [27], probably due to an increased number of branches. Heating to 80 °C leads to the disappearance of wide-angle reflections and a shift in the small-angle peak that corresponds to the transition to a smectic A (SmA) with a period of 28.7 Å. A decrease in the smectic period with the loss of order is characteristic of most semi-crystalline materials. The transition to an isotropic melt is observed at 118 °C.

**Figure 7:**
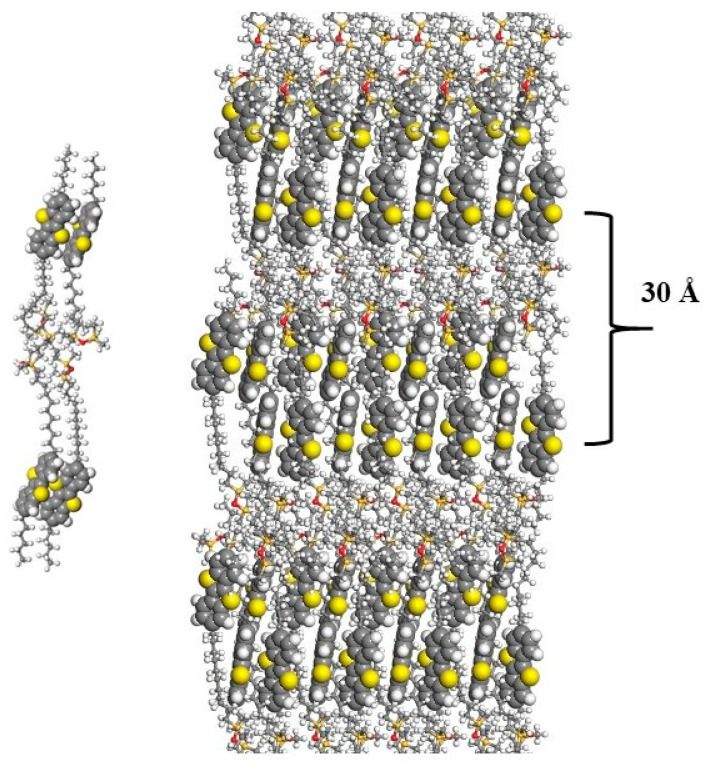
Molecular model of the compound **D4-Und-BTBT-Hex** in the extended conformation and in smectic E phase.

For the sample **D4-Hex-BTBT-Oct**, which has shorter spacers but longer terminal alkyl fragments, an intense small-angle reflection with an interplanar distance of 27.3 Å is also observed. At the same time, in the wide-angle region, reflections similar to those for **D4-Und-BTBT-Hex** are very weakly expressed, characterized by a large half-width, which indicates a small crystallite size, and finally disappear upon heating to 40 °C, which corresponds to the melting of the crystal lattice of BTBT fragments and the transition to smectic A. In this case, there is no change in the parameter of the liquid crystal packing until the transition to an isotropic melt at 117 °C. The interplanar distance corresponding to the small-angle reflection is 30 Å, which is close to the extended conformation of **D4-Und-BTBT-Hex** and indicates a structure of smectic ordering of the E type (Figure 7).

Thus, based on the structural data obtained, it can be concluded that the crystal packing is significantly disrupted, with a decrease in the spacer and an increase in the terminal fragments. Probably, the first factor has a determining influence, due to the constraint of free rotation during the ordering of benzothiophene fragments in the lattice, as well as the presence of a well-defined smectic E for the other compounds with BTBT groups and alkyl terminal substituents [35]. At the same time, the temperature stability and the mesophase parameter are preserved when modifying the length of the alkyl fragments.

### 2.4. Electrical Characteristics

The tetramers obtained, **D4-Und-BTBT-Hex** and **D4-Hex-BTBT-Oct**, were tested as active layers for OFETs, since the previously reported siloxane dimers, i.e., **D2-Und-BTBT-Hex**, **D2-Hex-BTBT-Hex** and **D2-Hept-BTBT-Hex** containing both an organosilicon component and a BTBT fragment, have demonstrated good semiconductor characteristics in OFETs [26,28]. The advantages of using organosilicon derivatives of BTBT are their good film forming properties and stability under normal conditions [36].

**Figure 8:**
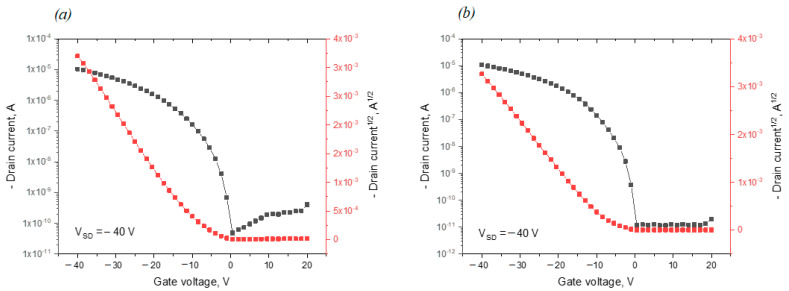
Transfer characteristics of the best devices for **D4-Und-BTBT-Hex** coupling in logarithmic coordinates (black) and as a function of the square root of the current (red) for concentrations: (**a**) 1 g L^−1^; (**b**) 2 g L^−1^. In turn, films based on the **D4-Hex-BTBT-Oct** compound demonstrated very weak semiconducting properties at high threshold voltages (Table 1). This can be explained by the fact that compound **D4-Und-BTBT-Hex** has a denser packing due to the longer length of the undecyl spacer between the organosilicon and BTBT fragments, which at room temperature forms an ordered SmE phase, as opposed to compound **D4-Hex-BTBT-Oct**, which forms a disordered SmA phase only.

Thin films of the **D4-Und-BTBT-Hex** and **D4-Hex-BTBT-Oct** compounds were obtained on a Si/SiO_2_ substrate by the spin-coating method. To form an even layer, the two-stage processing method was chosen: 500 rpm for 30 s and 1000 rpm for 90 s [26], varying the solution concentration—1.0 or 2.0 gL^−1^ (Table 1). The films were obtained for both compounds, **D4-Und-BTBT-Hex** and **D4-Hex-BTBT-Oct** (Figures S31–S37, ESI). The films’ thicknesses for both compounds ranged from 6 to 24 nm, which represents a few layers. However, good semiconducting properties were only recorded for the **D4-Und-BTBT-Hex** compound (Table 1, Figure 8).

The devices fabricated using the **D4-Und-BTBT-Hex** compound showed reproducible electrical characteristics, with the effective charge carrier mobility of the order of 10^−2^–10^−4^ cm^2^ V^−1^ s^−1^, a low threshold voltage and a high on/off ratio of 10^4^−10^7^ (Table 1 and Table S3, ESI). These data are comparable to or even better than the results obtained previously for siloxane dimers **D2-Und-BTBT-Hex**, **D2-Hex-BTBT-Hex** and **D2-Hept-BTBT-Hex** of similar structure under similar preparation conditions [26,28] (Table S3, ESI). Moreover, since a reliability factor r_sat_ [37] for the calculated mobility values is ca. 0.6, the effective mobility values presented in Table 1 are ca. 1.5 times less than the ones measured and reported for the dimers. It is worth noting that, in the case of tetramer **D4-Und-BTBT-Hex**, changing the solution concentration did not lead to a significant change in its electrical characteristics, unlike the dimer **D2-Und-BTBT-Hex**. The best characteristics for **D4-Und-BTBT-Hex** were obtained for batch 2, which showed less dewetting of organic semiconductor film from the substrate (compare Figures S31 and S32, ESI). Similar findings were observed for thin films of **D4-Hex-BTBT-Oct**, where only batch 2 showed less dewetting of the film with semiconductors characteristics, while batch 1 did not work in the OFETs (compare Figures S33 and S34, ESI). Still further optimization should be possible to reach the mobility values > 0.1 cm^2^ V^−1^ s^−1^, at least for **D4-Und-BTBT-Hex**, i.e., as had been achieved previously for **D2-Und-BTBT-Hex** using a PMMA interlayer on the dielectrics, where μ_max_ up to 0.47 cm^2^ V^−1^ s^−1^ was measured [26]. AFM topography images of the thin films surfaces and them profiles for **D4-Und-BTBT-Hex** and **D4-Hex-BTBT-Oct** see Figures S35–S37, ESI.

## 3. Materials and Methods

### 3.1. Materials

Commercially available reagents were used in this work: octanoyl chloride (Sigma-Aldrich, Burlington, MA, USA, 99%), hexanoyl chloride (Sigma-Aldrich, 97%), tetraallylsilane (Sigma-Aldrich, 97%), aluminum chloride (Sigma-Aldrich, 99%), zinc powder (Sigma-Aldrich, 99%), lithium aluminum hydride (Thermo Scientific, Waltham, MA, USA, 98%), glacial acetic acid (JSC “Base No. 1 of Chemical Reactants” Moscow, Russia), Karstedt catalyst (“abcr”, Karlsruhe, Germany, 3–3.5% Pt), 1,1,3,3-tetramethyldisiloxane (Sigma-Aldrich, 97%) and anhydrous sodium sulfate (JSC “Lenreaktiv”, Saint-Petersburg, Russia, chemically pure). Dry toluene (10 ppm) (JSC Vekton, Moscow, Russia, analytical grade), dry dichloromethane (20 ppm) (JSC Vekton, analytical grade), dry tetrahydrofuran (20 ppm) (ECOS-1, Moscow, Russia, chemically pure), cyclohexane (ECOS-1, analytical grade), diethyl ether (JSC Vekton, analytical grade), acetone (ECOS-1, analytical grade) and isopropanol (Reaktiv-express, Moscow, Russia, analytical grade) were used as solvents. All solvents were dried using standard methods; water content was monitored using a K. Fischer 831 KF Coulometer titrator (Metrohm, Herisau, Switzerland). Ultrapure deionized water with a resistance of 18 MΩ*cm was obtained using an Aquilon D-301 deionizer (Podolsk, Russia).

### 3.2. NMR-Spectroscopy

^1^H NMR spectra were recorded on a Bruker WP-250 SY spectrometer (Billerica, MA, USA) at a frequency of 250.13 MHz using the CDCl_3_ signal (7.25 ppm) as an internal standard. ^13^C and ^29^Si NMR spectra were recorded on a Bruker Avance II 300 spectrometer at a frequency of 75 MHz.

For ^1^H NMR spectroscopy, 2% solutions of the analyzed substances were prepared, for ^13^C and ^29^Si NMR spectroscopy—5% solutions, the results were processed on a computer using special ACDLabs 12.0 software.

### 3.3. Thermal Analysis

Thermogravimetric analysis of the samples was carried out in a dynamic mode in the range from 20 to 700 °C using the STA JUPITER 443 F3 NETZSCH system (Selb, Germany) with an accuracy of sample weight determination up to 1 mg. The heating rate was 10 deg/min in an atmosphere of air and argon.

DSC of the samples was carried out using a Perkin-Elmer DSC7 (Shelton, CT, USA) differential scanning calorimeter in a helium flow (30 mL min^−1^). Sample weight was about 10 mg. Weighing was performed using a Perkin-Elmer AD-4 Autobalance with an accuracy of 10^−5^ g. The samples were heated with a heating rate of 20 deg min^−1^, then cooled with the same rate and heated again.

### 3.4. X-Ray Diffraction Analysis

Small-angle and wide-angle X-ray diffraction analysis (XRD) of the samples was performed at the BioMUR station of the Kurchatov synchrotron (Moscow, Russia). The radiation source was a 1.7 T bending magnet with an energy of 8 keV (1.435 Å) with a resolution of dE/E 10^−3^ and a photon flux of 10^9^. The beam size on the sample was 0.5 × 0.3 mm^2^, and a Dectris Pilatus 1 M two-dimensional detector (DECTRIS Ltd., Baden-Daettwil, Switzerland) was used to record diffraction patterns. The sample-detector distance was 150 mm, and background scattering was subtracted before subsequent processing. Silver behenate and NaC (Na_2_Ca_3_A_l2_F_14_) were used as calibration standards at small and large angles. The range of backscattering vector q was 0.8–33 nm^−1^. A Linkam THMS 600 heating stage (Linkam Scientific Instruments Ltd., Redhill, Surrey, UK) with a heating rate of 7 deg min^−1^ was used to heat the sample in situ. The exposure time was 23 s; the samples were placed in Hilgenberg X-ray capillaries with a diameter of 2 mm and a wall thickness of 0.01 mm (Hilgenberg GmbH, Malsfeld, Germany). The open software packages Fit2D 18.0 (ESRF, Grenoble, France) and ImageJ 1.52u (NIH, Washington, D.C.,USA) were used to process the obtained scattering patterns.

### 3.5. Chromatographic Methods

Analysis by GPC was carried out on a chromatographic system: Shimadzu (Kyoto, Japan) with a RID10AVP refractometer, SPD-M10AVP diode array, 300 mm long and 7.8 mm in diameter (300 × 7.8 mm^2^) columns (Phenomenex, Torrance, CA, USA) filled with the Phenogel sorbent (Phenomenex, USA), pore size 500 Å, thermostatting temperature—40 °C ± 0.1 °C, eluent—tetrahydrofuran (THF). The system consisted of a STAYER series 2 high-pressure pump (Aquilon, Russia), a SmartlineRI 2300 refractometric detector (KNAUER, Berlin, Germany) and a JETSTREAM 2 PLUS column thermostat (KNAUER, Germany). Thermostat temperature 40 °C ± 0.1 °C, eluent—THF, flow rate—1.0 mL/min, columns 300 mm long and 7.8 mm in diameter (300 × 7.8 mm^2^) filled with Phenogel sorbent (Phenomenex, USA), particle size—5 μm, pore size 104 Å. The analysis results were processed using the MultiChrom 1.6 GPC program (Ampersend, Krasnogorsk, Russia) using polystyrene standards.

For preparative separation of the mixture, a PuriFlash XS 520Plus chromatograph (Interchim, Montleçon, France) with a PF-XS520Plus 200–400 nm UV detector, a panVap-6 four-channel air pump and PF-50SIHP-00120 columns filled with Silica Inorganic Sorbent (Sigma-Aldrich, USA), pore size 50 μm were used.

### 3.6. Mass-Spectrometry MALDI

Analysis by MALDI was carried out on mass spectrometer “Autobio Autof MS 1600” (Autobio Diagnostics Co., Zhengzhou, China). Samples were dissolved in toluene (for spectroscopy “Component reactive”, Mocsow, Russia) to a concentration of 12 mg/mL. DCTB (trans-2-[3-(4-tert-butylphenyl)-2-propenylidene]malononitrile with a concentration of 20 mg/mL in propanol) was used as a matrix. The probe (volume 3 μL) was applied layer by layer and dried at 25 °C. Positive ions were recorded in a linear mode in the range of 200–4000 Da. External calibration was performed using polyethylene glycol standards in the range of 480–3860 Da.

### 3.7. Laboratory Equipment for Synthesis

Microwave organic synthesis system “CEM Discovery” (Matthews, NC, USA) was used to synthesize compounds **6a**, **b** at 100 °C and 50 W power for 15 min.

### 3.8. Synthesis Methods

Compounds **1**–**7** were obtained according to the literature methods: compound **1** [38]; compounds **2**–**7** [25,32].

**5,6-dibromo-1-(7-octyl[1]Benzothieno[3,2-b]benzothiophen-2-yl)hexan-1-one (4b).**

The yield was 5.01 g (68%). ^1^H NMR spectrum (CDCl_3_) δ ppm: 8.53 (d, *J* = 1.22 Hz, 1H), 8.02–8.08 (m, 1H), 7.73–7.93 (m, 3H), 7.27–7.34 (m, 1H), 4.16–4.29 (m, 1H), 3.84–3.93 (m, 1H), 3.62–3.73 (m, 1H), 3.09–3.19 (m, 2H), 2.76 (t, *J* = 7.78 Hz, 2H), 1.88–2.37 (m, 4H), 1.68 (d, *J* = 7.02 Hz, 2H), 1.22–1.42 (m, 10H), 0.83–0.93 (m, 3H). ^13^C NMR spectrum (CDCl_3_) δ ppm: 198.10, 151.12, 146.23, 139.72, 137.28, 136.32, 133.74, 124.82, 123.12, 122.54, 120.22, 53.96, 42.07, 37.21, 36.71, 32.26, 29.62, 29.58, 22.76, 20.18, 13.23. Found, %: C 55.14; H 5.28; S 10.58. Calculated, %: C 55.27; H 5.30; S 10.54.

**2-(5,6-dibromohexyl)-7-octyl[1]Benzothieno[3,2-b]benzothiophene (5b).**

The yield was 3.76 g (77%). ^1^H NMR spectrum (CDCl_3_) δ ppm: 7.69–7.80 (m, 4H), 7.23–7.28 (m, 2H), 4.10–4.24 (m, 1H), 3.85 (dd, *J*_1_ = 10.38 Hz, *J*_2_ = 4.27 Hz, 1H), 3.56–3.68 (m, 1H), 2.70–2.84 (m, 4H), 2.13–2.29 (m, 1H), 1.61–1.93 (m, 7H), 1.23–1.42 (m, 10H), 0.83–0.93 (m, 3H). ^13^C NMR spectrum (CDCl_3_) δ ppm: 141.23, 138.46, 136.82, 124.72, 123.91, 122.42, 52.36, 38.48, 37.51, 35.86, 31.98, 31.26, 31.02, 29.60, 29.48, 25.41, 22.12, 13.26. Found, %: C 56.62; H 5.81; S 10.74. Calculated, %: C 56.57; H 5.76; S 10.79.

**2-(hex-5-en-1-yl)-7-octyl[1]Benzothieno[3,2-b]benzothiophene (6b).**

The yield was 2.46 g (91%). ^1^H NMR spectrum (CDCl_3_) δ ppm: 7.67–7.80 (m, 4H), 7.23–7.28 (m, 2H), 5.81 (ddt, *J*_1_ = 17.01, *J*_2_ = 10.30, *J*_3_ = 6.49 Hz, 1H), 4.91–5.07 (m, 2H), 2.75 (td, *J*_1_ = 7.55, *J*_2_ = 3.81 Hz, 4H), 2.04–2.18 (m, 2H), 1.62–1.79 (m, 4H), 1.42–1.58 (m, 3H), 1.23–1.40 (m, 10H), 0.82–0.92 (m, 3H). ^13^C NMR spectrum (CDCl_3_) δ ppm: 141.12, 139.46, 137.13, 136.82, 123.12, 122.43, 122.01, 116.03, 35.86, 34.13, 29.48, 28.92, 28.18, 22.12, 13.23. Found, %: C 77.52; H 7.79; S 14.69. Calculated, %: C 77.37; H 7.88; S 14.75.

**1,1,3,3-tetramethyl-1-(6-(7-octyl[1]Benzothieno[3,2-b]benzothiophen-2-yl)hexyl)disiloxane (7b).**

The yield was 2.99 g (95%). ^1^H NMR spectrum (CDCl_3_) δ ppm: 7.67–7.80 (m, 4H), 7.25–7.31 (m, 2H), 4.63–4.71 (m, 1H), 2.75 (t, *J* = 7.63 Hz, 4H), 1.66–1.71 (m, 4H), 1.22–1.40 (m, 16H), 0.82–0.93 (m, 3H), 0.54 (d, *J* = 8.85 Hz, 2H), 0.15 (d, *J* = 2.75 Hz, 6H), 0.02–0.09 (m, 6H). ^13^C NMR spectrum (CDCl_3_) δ ppm: 139.73, 137.29, 136.32, 123.12, 123.05, 122.52, 36.71, 35.86, 31.28, 29.62, 22.76, 19.83, 13.23, 5.96, 2.12. Found, %: C 67.52; H 8.36; S 11.21. Calculated, %: C 67.55; H 8.50; S 11.27.

**General procedure for the synthesis of compounds D4-Und-BTBT-Hex, D4-Hex-BTBT-Oct.**

A single-neck flask (10 mL), a reflux condenser and an argon inlet/outlet valve were dried at 150 °C for 1 h. The reaction apparatus was assembled and cooled in an argon stream. Tetraallylsilane (1 equiv.) and a solution of compound **7** (5 equiv.) in a minimum volume of dry toluene (11 ppm) were added to the reaction flask. Then, 30 mol% of Karstedt’s catalyst was added to the reaction flask and stirred while heating at 70 °C for 6 h. The reaction was monitored by GPC and TLC (eluent cyclohexane/toluene 3%). The resulting reaction mixture was evaporated on a vacuum rotary evaporator. Purification was carried out by column chromatography on silica gel, eluent cyclohexane/toluene 10%.

***Tetrakis*(3-(1,1,3,3-tetramethyl-1-(11-(7-hexyl[1]Benzothieno[3,2-b]benzothiophen-2-yl)undecyl)disiloxanyl)propyl)silane (D4-Und-BTBT-Hex).**

From 0.81 g (1.33 mmol) of compound **7a**, 0.053 g (0.27 mmol) of tetraallylsilane, 65 μL (0.12 mmol) of Karstedt catalyst and 5 mL of toluene, 0.28 g (38%, purity 97%) of substance **D4-Und-BTBT-Hex** was obtained as a white powder. ^1^H NMR spectrum (CDCl_3_) δ ppm: 7.64–7.79 (m, 16H), 7.21–7.30 (m, 8H), 2.68–2.81 (m, 16H), 1.67 (d, *J* = 7.15 Hz, 16H), 1.30 (d, *J* = 19.07 Hz, 96H), 0.84–0.95 (m, 12H), 0.46–0.66 (m, 24H), 0.04 (s, 48H). ^13^C NMR spectrum (CDCl_3_) δ ppm: 142.35, 140.00, 132.49, 131.14, 125.77, 123.26, 121.01, 36.10, 33.51, 31.72, 31.67, 29.75, 29.66, 29.57, 29.45, 29.37, 28.98, 23.42, 23.32, 22.60, 18.43, 17.98, 17.19, 14.11, 0.51, 0.43. ^29^Si NMR spectrum (CDCl_3_) δ ppm: 7.30, 6.72. Found, %: C 69.28; H 9.04; S 9.65. Calculated, %: C 69.24; H 9.02; S 9.73. MALDI (*m*/*z*): found [M − H^+^]:2632.3052; calculated [M − H^+^]: 2632.3874.

***Tetrakis*(3-(1,1,3,3-tetramethyl-1-(6-(7-octyl[1]Benzothieno[3,2-b]benzothiophen-2-yl)hexyl)disiloxanyl)propyl)silane (D4-Hex-BTBT-Oct).**

From 1.20 g (2.11 mmol) of compound **7b**, 0.081 g (0.42 mmol) of tetraallylsilane, 100 μL (0.18 mmol) of Karstedt’s catalyst and 5 mL of toluene, 0.24 g (28%, purity 99%) of substance **D4-Hex-BTBT-Oct** was obtained as a white powder. ^1^H NMR spectrum (CDCl_3_) δ ppm: 7.63–7.76 (m, 16H), 7.22 (m, 8H), 2.72 (m, 16H), 1.61–1.70 (m, 16H), 1.30 (d, *J* = 17.70 Hz, 72H), 0.83–0.93 (m, 12H), 0.44–0.63 (m, 24H), 0.02–0.08 (m, 48H). ^13^C NMR spectrum (CDCl_3_) δ ppm: 142.12, 140.03, 132.54, 131.24, 125.79, 123.37, 121.06, 35.98, 33.61, 31.92, 31.57, 29.78, 29.56, 29.44, 22.71, 22.43, 20.02, 19.19, 13.21, 0.54, 0.40. ^29^Si NMR spectrum (CDCl3) δ ppm: 7.31, 6.72. Found, %: C 68.09; H 8.76; S 10.42. Calculated, %: C 68.12; H 8.66; S 10.39. MALDI (*m*/*z*): found [M + H^+^]:2466.3345; calculated [M + H^+^]: 2466.2153.

### 3.9. OFET Architecture

OFETs were fabricated on 20 mm × 15 mm Si/SiO_2_ substrates pre-washed in piranha, acetone and isopropanol. Highly doped silicon wafers with thermally grown 300 nm thick silicon dioxide layers were used as the gate electrode and dielectric (dielectric capacitance 11.5 nFcm^−2^). The bottom electrodes “source” and “drain” were fabricated by thermal evaporation (Au, 40 nm) on the substrates through shadow masks with gold electrodes in the bottom contact geometry, and had the following geometric dimensions: channel length L = 30 µm and channel width W = 1000 µm (W/L ratio = 33).

### 3.10. Surface Preparation

Before deposition of the semiconductor layer, the substrates were plasma-treated using a low-pressure plasma system Diener Electronic (Diener Electronic GmbH, Ebhausen, Germany) at a frequency of 40 kHz and a power of 200 W. The contacts were modified in a solution of 2,3,4,5,6-pentafluorobenzenethiol (PFBT) (Sigma Aldrich Co., USA), according to the literature method [39].

### 3.11. Application Method

The spin-coating method was chosen as a method for producing the active semiconductor layer of OFETs using a WS-650MZ-8NPP/UD3 setup (Laurell Technologies Corporation, Lansdale, PA, USA). The deposition solution was prepared by dissolving the tetramers in toluene at a concentration of 1.0 and 2.0 g L^−1^. The active layer was deposited under the following conditions: step 1—acceleration 500 rpm, rotation speed 750 rpm, rotation time 30 s; step 2—1000 rpm, rotation time 90 s. Dropping volume—50 μL.

### 3.12. Film Characterization

The morphology of thin films was studied using an NT-MDT atomic-force microscope (SOLVER NEXT, Zelenograd, Russia) in tapping mode under ambient conditions using NT-MDT HA-FM silicon probes with a resonance frequency of 77 kHz. Optical micrographs were obtained on an Axioscop A40Pol polarization optical microscope (Carl Zeiss, Oberkochen, Germany).

### 3.13. Electrical Measurements

The current-voltage characteristics were measured in air on a probe station (ProbeStation 100, Printeltech, Moscow, Russia) using a Keithley 2634B source-measure unit (Tektronix, Beaverton, OR, USA). To avoid current leakage, the perimeter of the active zone of the device was carefully scratched. The charge carrier mobility and threshold voltages were estimated according to the Shockley model [40]. Since nonlinearities were observed in the OFET characteristics, the effective mobility μ_eff_. was calculated based on the reliability factor r_sat_ introduced by V. Podzorov et al. [37] and described in [39].

## 4. Conclusions

In this work, two novel organosilicon tetramers with dialkyl-substituted [1]Benzothieno[3,2-b]benzothiophene moieties, **D4-Und-BTBT-Hex** and **D4-Hex-BTBT-Oct**, were synthesized and investigated. The target tetramers were obtained by the hydrosilylation reaction between tetraallylsilane and 1,1,3,3-tetramethyl-1-(ω-(7-alkyl[1]Benzothieno[3,2-b]benzothiophen-2-yl)alkyl)disiloxanes in 28–38% yields. The chemical structure of the compounds obtained was confirmed by ^1^H, ^13^C and ^29^Si NMR spectroscopy, gel permeation chromatography and elemental analysis. The phase behavior of these compounds was studied by DSC, POM and XRD. It was shown that, in compound **D4-Und-BTBT-Hex**, there are two phase transitions which correspond to phase transitions from smectic E to smectic A with subsequent isotropization. In turn, for compound **D4-Hex-BTBT-Oct**, wide-angle reflections are weakly expressed, characterized by a large half-width, which indicates a small crystallite size and, up to 40 °C, a phase transition to smectic A occurs with subsequent isotropization at 118 °C. A significant disruption of the crystal packing in the obtained tetramers occurs, with a decrease in the spacer length and an increase in the terminal fragments. Temperature stability and mesophase parameters are maintained, with a change in the length of the alkyl moieties. The investigation of tetrameric structures **D4-Und-BTBT-Hex** and **D4-Hex-BTBT-Oct** as an active layer in OFETs showed that the tetramer with the longer undecyl spacer length and the shorter hexyl terminal groups **D4-Und-BTBT-Hex** has demonstrated good electrical properties, with a maximum charge mobility of up to 3.5 × 10^−2^ cm^2^ V^−1^ s^−1^, a low threshold voltage and an on/off ratio up to 10^7^. This is comparable or even better for the semiconductor properties of the similar disiloxane dimer **D2-Und-BTBT-Hex**, measured previously under similar conditions. However, a tetramer with a shorter hexyl spacer and even longer alkyl end groups, **D4-Hex-BTBT-Oct**, exhibited weak semiconducting properties in OFETs, consistent with DSC and X-ray diffraction data, indicating its lower order at room temperature. These results pave the way for the development of more advanced soluble organic semiconductor solutions with pronounced semiconducting properties.

## Figures and Tables

**Table 1:** Electrical performance data for the OFETs based on different organic semiconductors prepared at different concentrations.

Organic Semiconductor	C, g L^−1^	μ_eff.max_ (µ_eff.ave_), cm^2^ V^−1^ s^−1^	Vth, V	I_on/off_	Number of Devices	Thickness (nm)
**D4-Und-BTBT-Hex**	1.0	1.6 × 10^−3^ (5.2 × 10^−4^ ± 3.2 × 10^−4^)	−5.3 to 4.9	10^5^−10^7^	19 *	7–16
	2.0	1.8 × 10^−3^ (8.0 × 10^−4^ ± 4.4 × 10^−4^)	−5.8 to 13.1	10^4^−10^7^	19 *	5–14
	1.0	3.3 × 10^−2^ (2.0 × 10^−2^ ± 1.0 × 10^−2^)	−20.1 to −4.0	10^3^–10^5^	14 **	6–10
	2.0	3.5 × 10^−2^ (2.1 × 10^−2^ ± 6.7 × 10^−3^)	−17.1 to −7.0	10^3^–10^5^	18 **	6–24
**D4-Hex-BTBT-Oct**	1.0	3.8 × 10^−6^ (2.6 × 10^−6^ ± 8.8 × 10^−7^)	−11.0 to +14.0	10^0^−10^1^	5 **	6–9
	2.0	4.5 × 10^−6^ (3.4 × 10^−6^ ± 7.1 × 10^−7^)	−11.0 to +2.0	10^0^−10^1^	19 **	9–10

Notes. C—concentration of the initial solution for obtaining active layers, μ_eff.max_ (µ_eff.ave_)—effective average (maximum) mobility of charge carriers, Vth—threshold voltage, I_on/off_—ratio of currents in the on and off state, *—batch 1, **—batch 2.

## Data Availability

Supplementary data associated with this article can be found in the online version at [reference to Supplementary Information of this article].

## Supplementary Materials

The following supporting information can be downloaded at https://www.mdpi.com/article/10.3390/molecules30234639/s1, Figure S1: ^1^H NMR spectra of compound **1**; Figure S2: ^1^H NMR spectra of compound **2a**; Figure S3: ^1^H NMR spectra of compound **2b**; Figure S4: ^1^H NMR spectra of compound **3a**; Figure S5: ^1^H NMR spectra of compound **3b**; Figure S6: ^1^H NMR spectra of compound **4a**; Figure S7: ^1^H NMR spectra of compound **4b**; Figure S8: ^13^C NMR spectra of compound **4b**; Figure S9: ^1^H NMR spectra of compound **5a**; Figure S10: ^1^H NMR spectra of compound **5b**; Figure S11: ^13^C NMR spectra of compound **5b**; Figure S12: ^1^H NMR spectra of compound **6a**; Figure S13: ^1^H NMR spectra of compound **6b**; Figure S14: ^13^C NMR spectra of compound **6b**; Figure S15: ^1^H NMR spectra of compound **7a**; Figure S16: ^1^H NMR spectra of compound **7b**; Figure S17: ^13^C NMR spectra of compound **7b**; Figure S18: ^1^H NMR spectra of compound **D4-Und-BTBT-Hex**; Figure S19: ^13^C NMR spectra of compound **D4-Und-BTBT-Hex**; Figure S20: ^29^Si NMR spectra of compound **D4-Und-BTBT-Hex**; Figure S21: ^1^H NMR spectra of compound **D4-Hex-BTBT-Oct**; Figure S22: ^13^C NMR spectra of compound **D4-Hex-BTBT-Oct**; Figure S23: ^29^Si NMR spectra of compound **D4-Hex-BTBT-Oct**; Figure S24: GPC curve of compound **7a**; Figure S25: GPC curve of compound **D4-Und-BTBT-Hex**; Figure S26: GPC curve of compound **7b**; Figure S27: GPC curve of compound **D4-Hex-BTBT-Oct**; Figure S28: Mass spectrum of the compound **D4-Und-BTBT-Hex**; Figure S29: Mass spectrum of the compound **D4-Hex-BTBT-Oct**; Table S1: Temperatures and enthalpies of phase transitions; Figure S30: Diffraction patterns during heating in situ in an X-ray beam at a rate of 6 °C/min: (a) sample **D4-Und-BTBT-Hex**; (b) sample **D4-Hex-BTBT-Oct**; Table S2: Original data for electrical characteristics of the devices (batch 2); Table S3: Electrical performance dataset for BTBT-based OFETs fabricated under different conditions applied by spin-coating method; Figure S31: Optical micrographs of **D4-Und-BTBT-Hex** thin films obtained from the solutions with a concentration of 1.0 g L^−1^ (a–c) and with a concentration of 2.0 g L^−1^ (d–f) in cross polarizers (a,d), in dark field (b,e) and in bright field (c,f); batch 2; Figure S32: Optical micrographs of **D4-Und-BTBT-Hex** thin films obtained from the solutions with a concentration of 1.0 g L^−1^ (a–c) and with a concentration of 2.0 g L^−1^ (d–f) in cross polarizers (a,d), in dark field (b,e) and in bright field (c,f), batch 1; Figure S33: Optical micrographs of **D4-Hex-BTBT-Oct** thin films obtained from the solutions with a concentration of 1.0 g L^−1^ (g–i) and with a concentration of 2.0 g L^−1^ (j–l) in cross polarizers (g,j), in dark field (h,k) and in bright field (i,l), batch 2; Figure S34: Optical micrographs of **D4-Hex-BTBT-Oct** thin films obtained from the solutions with a concentration of 1.0 g L^−1^ (g–i) and with a concentration of 2.0 g L^−1^ (j–l) in cross polarizers (g,j), in dark field (h,k) and in bright field (i,l), batch 1; Figure S35: AFM topography images of **D4-Und-BTBT-Hex** thin films surface obtained from the solutions with a concentration of 1.0 g L^−1^ (a) and with a concentration of 2.0 g L^−1^ (b) and corresponding cross-sections along horizontal gray lines in each image (c,d) (batch 2); Figure S36: AFM topography images of **D4-Hex-BTBT-Oct** thin films surface obtained from the solutions with a concentration of 1.0 g L^−1^ (a) and with a concentration of 2.0 g L^−1^ (b) and corresponding cross-sections along horizontal gray lines in each image (c,d) (batch 2); Figure S37: Thin film surface profiles of the AFM topography image fragments of **D4-Und-BTBT-Hex** (concentration of 1.0 g L^−1^) (a); of **D4-Hex-BTBT-Oct** (concentration of 1.0 g L^−1^) (b) (batch 2).

## Abbreviations

The following abbreviations are used in this manuscript:

BTBT	[1]Benzothieno[3,2-b]benzothiophene
NMR	Nuclear magnetic resonance
OFETs	Organic field-effect transistors
OSC	Organic semiconductor
OLET	Organic light-emitting transistor
OECT	Organic electrochemical transistor
OLED	Organic light-emitting diode
OPVs	Organic photovoltaics
TMDS	1,1,3,3-Tetramethyldisiloxane
GPC	Gel permeation chromatography
TLC	Thin-layer chromatography
TGA	Thermogravimetric analysis
POM	Polarization optical microscopy
DSC	Differential scanning calorimetry
XRD	X-ray diffraction analysis
AFM	Atomic-force microscope
