# Supplementary material for: Novel Organosilicon Tetramers with Dialkyl-Substituted [1]Benzothieno[3,2-b]benzothiophene Moieties for Solution-Processible Organic Electronics
Source: Molecules. 2025 Dec 3;30(23):4639. doi: 10.3390/molecules30234639 (PMC12693101; doi:10.3390/molecules30234639)
Supplement: Supplementary file 1 [file molecules-30-04639-s001.zip › molecules-3933355-supplementary.pdf]

Supporting information

Novel Organosilicon Tetramers with Dialkyl-Substituted [1]Benzothieno[3,2-*b*]benzothiophene Moieties for Solution-Processible Organic Electronics

Irina O. Gudkova [1,*], Evgeniy A. Zaborin [1], Alexander I. Buzin [1], Artem V. Bakirov [1,2,]
Yaroslava O. Titova [1], Oleg V. Borshchev [1], Sergey N. Chvalun [1,2] and Sergey A. Ponomarenko [1,*]

1 Enikolopov Institute of Synthetic Polymeric Materials of the Russian Academy of Sciences, Profsoyuznaya Str. 70, Moscow 117393, Russia
2 Kurchatov Institute National Research Center, 1 Akademika Kurchatova Sq., Moscow 123182, Russia
* Correspondence: i.gudkova@ispm.ru (I.O.G.); ponomarenko@ispm.ru (S.A.P.); Tel.: +7-(495)-332-58-83 (I.O.G.)

Contents:

1. NMR spectra Page **S2**

2. GPC curves Page **S19**

3. MALDI spectra Page **S21**

4. DSC data Page **S22**

5. X-ray diffraction Page **S23**

6. Electrical performance data for obtained OFETs Page **S24**

7. Micrographs of the OFETs film surface Page **S27**

8. AFM images of the OFETs surface Page **S29**

1. NMR spectra

^{1}H NMR spectrum ($CDCl_3$) δ ppm: 7.86-7.96 (m, 4H), 7.36-7.51 (m, 4H).

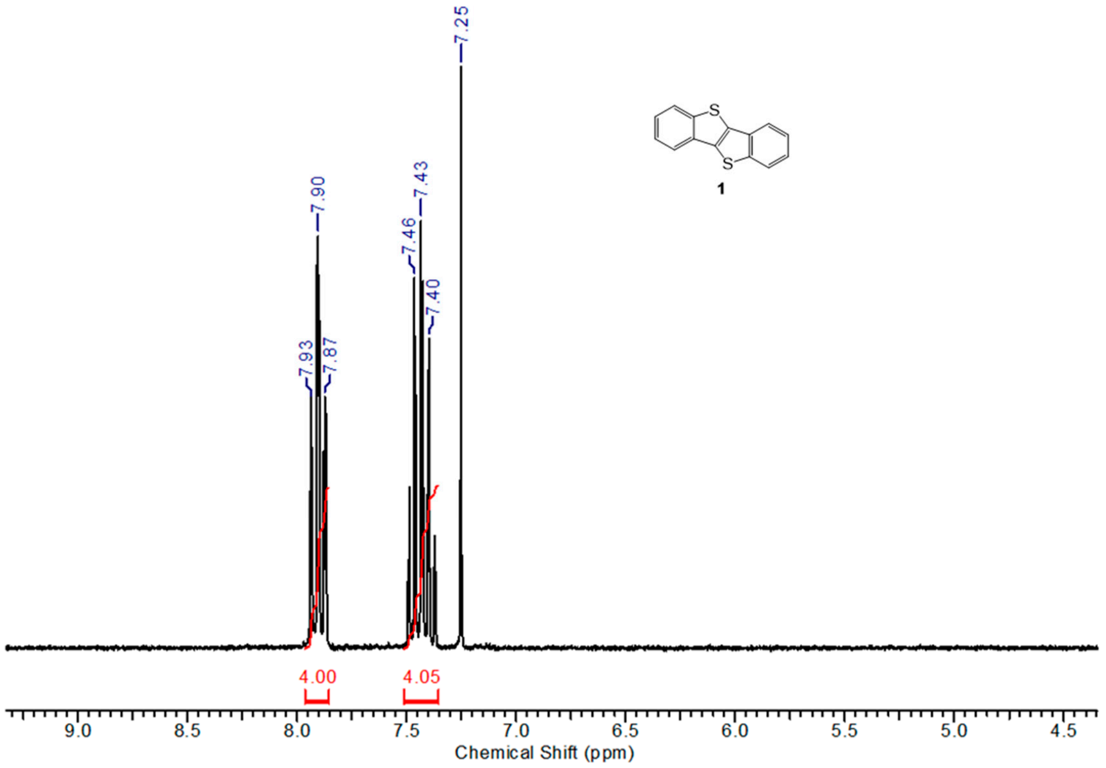

Figure S1. ^{1}H NMR spectra of compound **1**.

^{1}H NMR spectrum ($CDCl_3$) δ ppm: 8.54 (s, 1H), 8.02-8.11 (m, 1H), 7.89-8.00 (m, 3H), 7.42-7.48 (m, 2H), 3.03-3.07 (m, 2H), 1.78-1.82 (m, 2H), 1.40-1.44 (m, 4H), 0.93 (s, 3H).

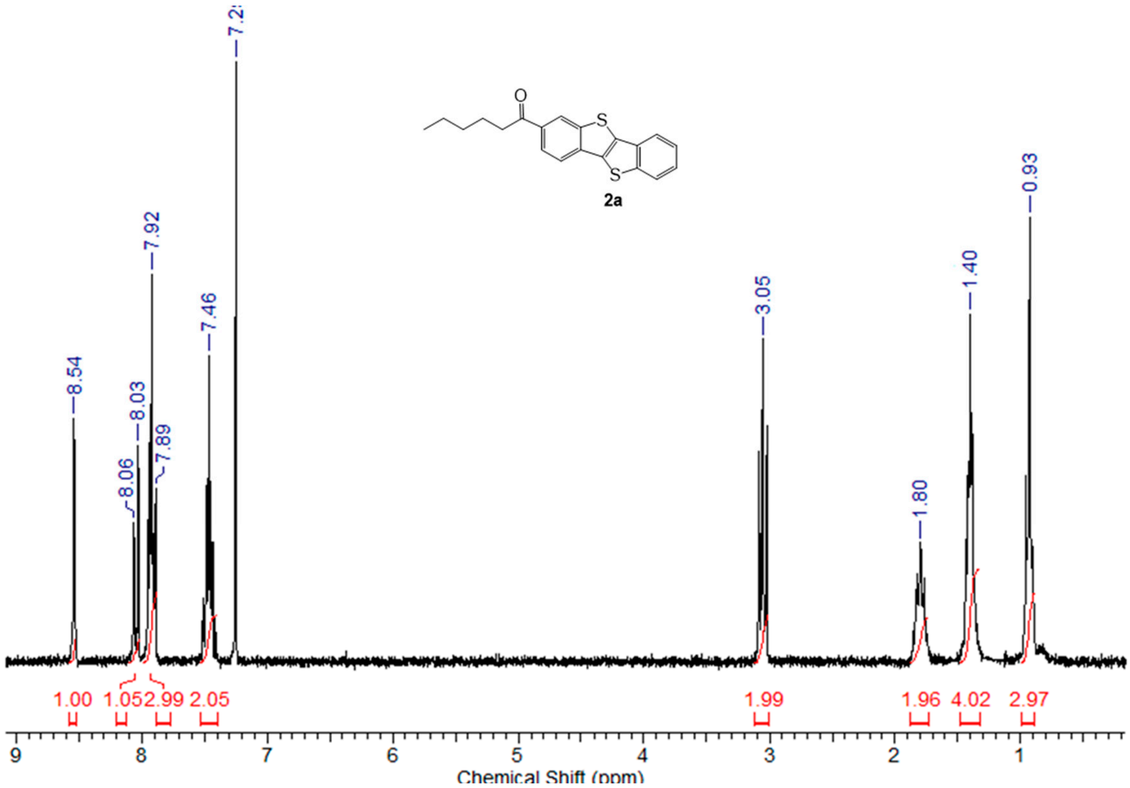

Figure S2. ^{1}H NMR spectra of compound **2a**.

^1H NMR spectrum ($CDCl_3$) δ ppm: 8.52 (c, 1H), 8.01-8.08 (m, 1H), 7.87-7.98 (m, 3H), 7.39 - 7.54 (m, 2H), 3.05 (t, J = 7.48 Hz, 2H), 1.78 (dt, J_1 = 14.80, J_2 = 7.25 Hz, 2H), 1.25-1.49 (m, 8H), 0.83-0.96 (m, 3H).

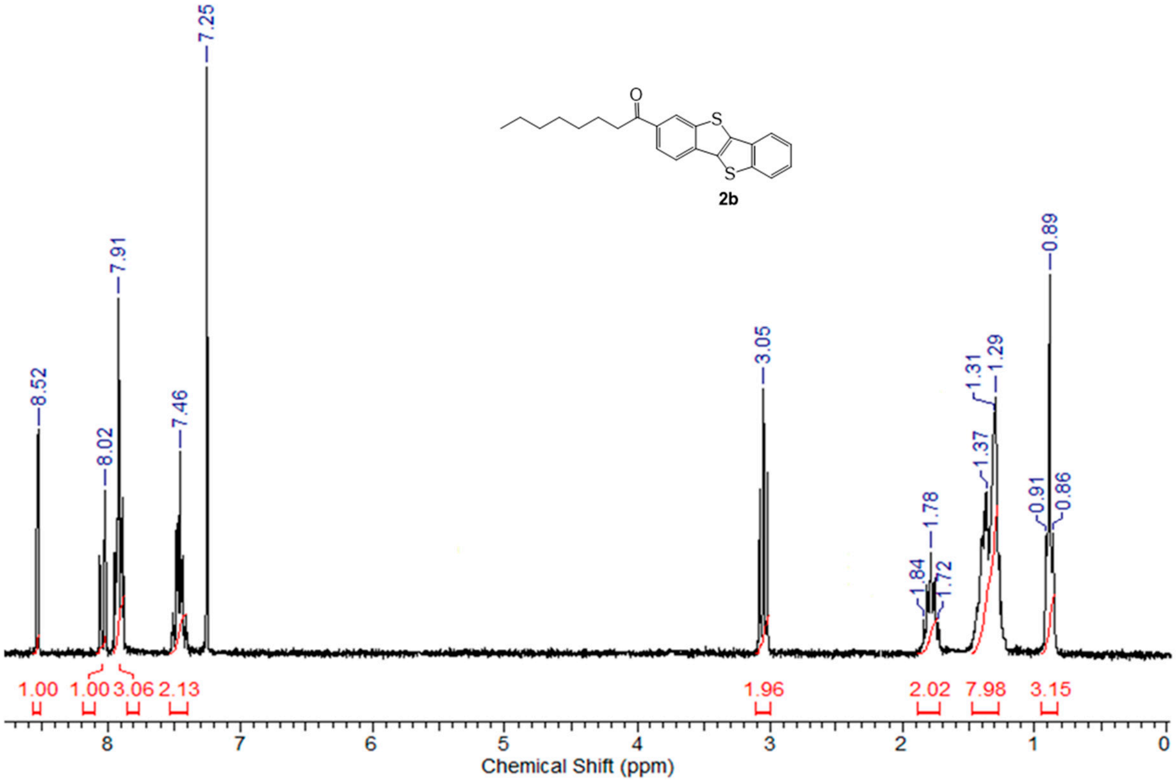

Figure S3. ^1H NMR spectra of compound **2b**.

^{1}H NMR spectrum ($CDCl_3$) δ ppm: 7.69-7.96 (m, 4H), 7.34-7.50 (m, 2H), 7.29 (d, *J* = 1.53 Hz, 1H), 2.72-2.77 (m, 2H), 1.62-1.73 (m, 2H), 1.32-1.35 (m, 6H), 0.84-0.95 (m, 3H).

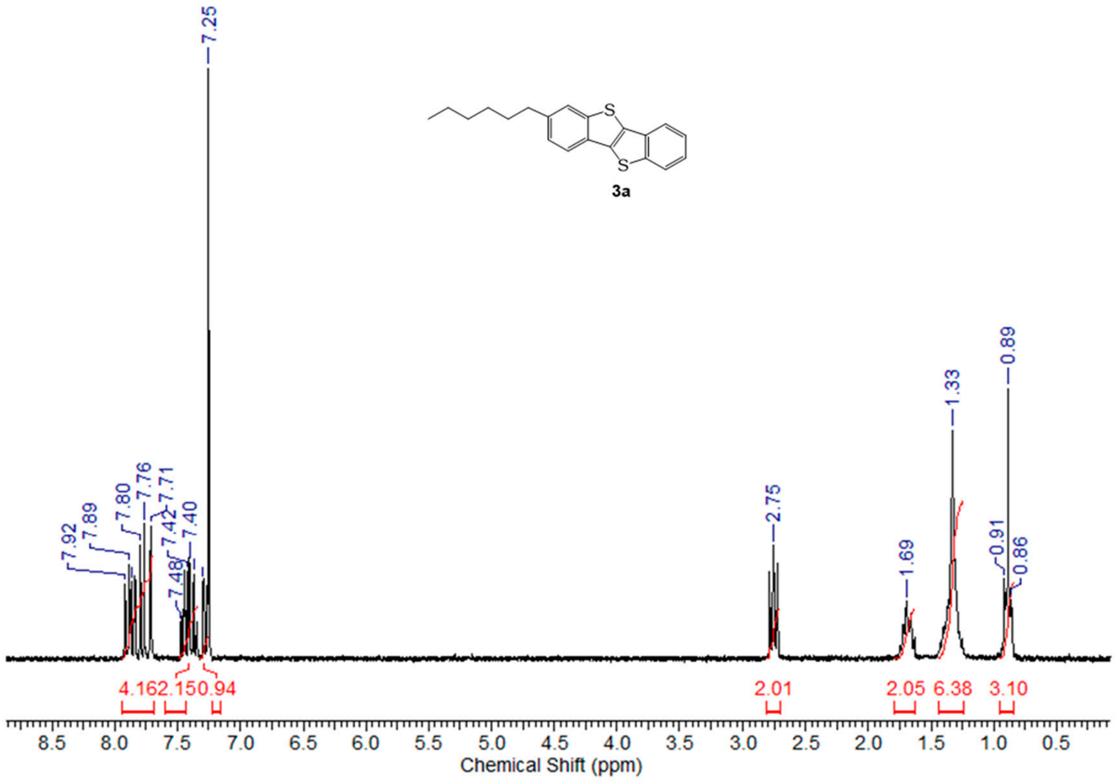

Figure S4. ^{1}H NMR spectra of compound **3a**.

^{1}H NMR spectrum ($CDCl_3$) δ ppm: 7.69-7.95 (m, 4H), 7.33-7.49 (m, 2H), 7.29 (d, *J* = 1.53 Hz, 1H), 2.69-2.82 (m, 2H), 1.68 (d, *J* = 7.63 Hz, 2H), 1.22-1.39 (m, 10H), 0.81-0.94 (m, 3H).

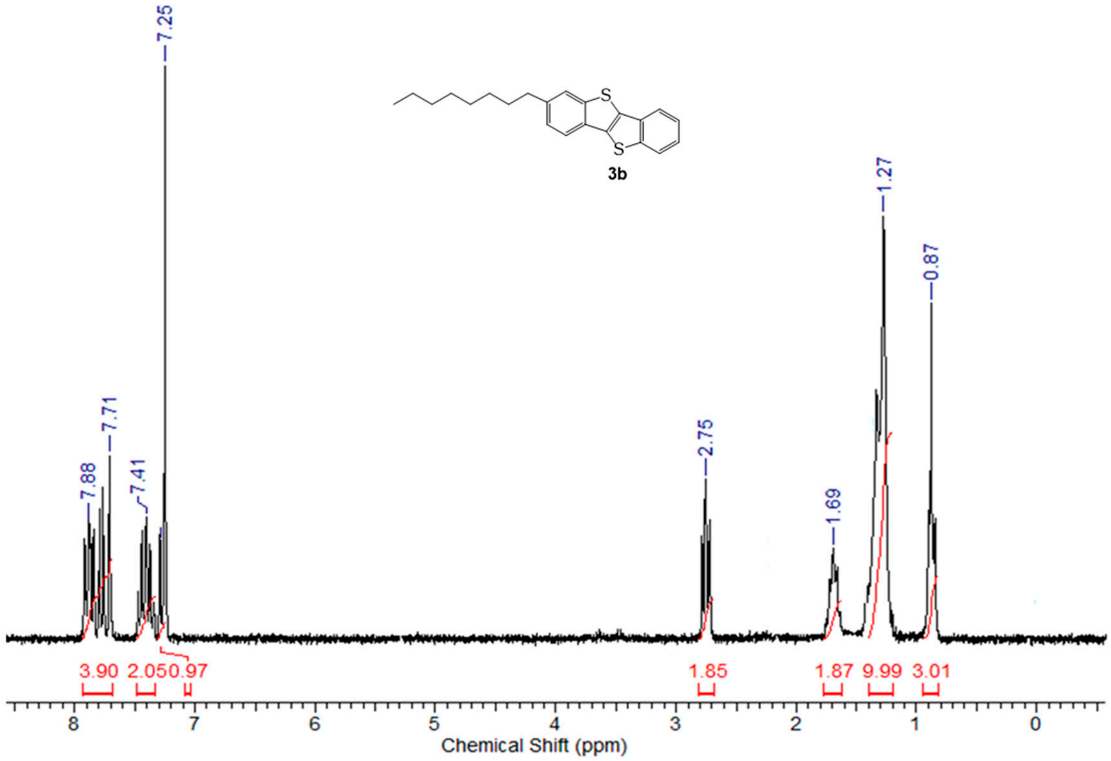

Figure S5. ^{1}H NMR spectra of compound **3b**.

^{1}H NMR spectrum ($CDCl_3$) δ ppm: 8.53 (s, 1H), 8.04 (dd, J_1 = 8.24, J_2 = 1.22 Hz, 1H), 7.75-7.95 (m, 2H), 7.73 (s, 1H), 7.30 (d, J = 8.24 Hz, 1H), 4.10-4.23 (m, 1H), 3.80-3.89 (m, 1H), 3.56-3.69 (m, 1H), 3.06 (t, J = 7.32 Hz, 2H), 2.77 (t, J = 7.63 Hz, 2H), 2.04-2.23 (m, 1H), 1.63-1.88 (m, 5H), 1.34 (d, J = 3.97 Hz, 16H), 0.84-0.95 (m, 3H).

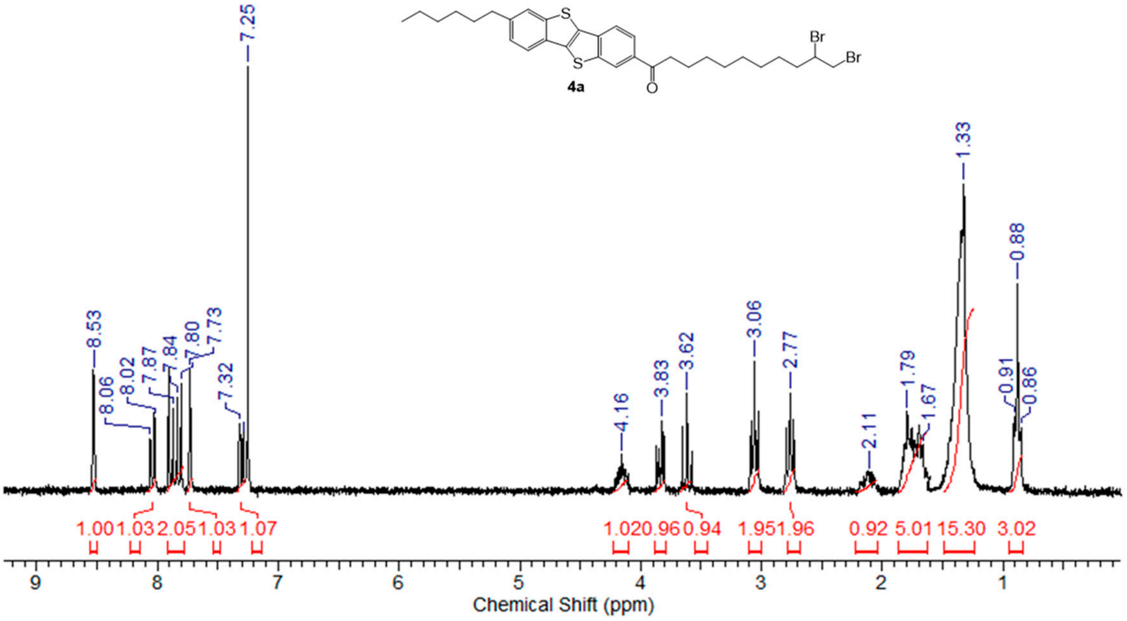

Figure S6. ^{1}H NMR spectra of compound **4a**.

¹H NMR spectrum ($CDCl_3$) δ ppm: 8.53 (d, *J* = 1.22 Hz, 1H), 8.02-8.08 (m, 1H), 7.73-7.93 (m, 3H), 7.27-7.34 (m, 1H), 4.16-4.29 (m, 1H), 3.84-3.93 (m, 1H), 3.62-3.73 (m, 1H), 3.09-3.19 (m, 2H), 2.76 (t, *J* = 7.78 Hz, 2H), 1.88-2.37 (m, 4H), 1.68 (d, *J* = 7.02 Hz, 2H), 1.22-1.42 (m, 10H), 0.83-0.93 (m, 3H).

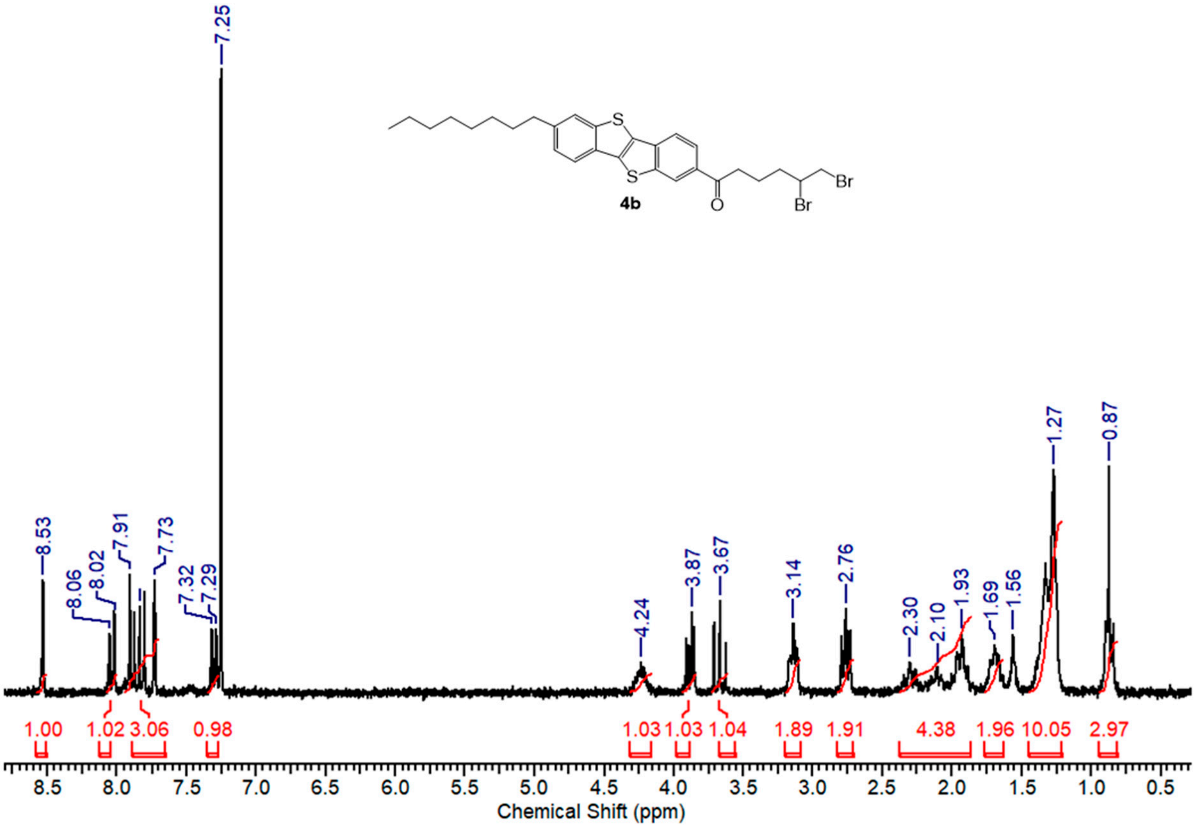

Figure S7. ¹H NMR spectra of compound **4b**.

¹³C NMR spectrum ($CDCl_3$) δ ppm: 198.10, 151.12, 146.23, 139.72, 137.28, 136.32, 133.74, 124.82, 123.12, 122.54, 120.22, 53.96, 42.07, 37.21, 36.24, 32.26, 29.62, 29.58, 22.76, 20.18, 13.23.

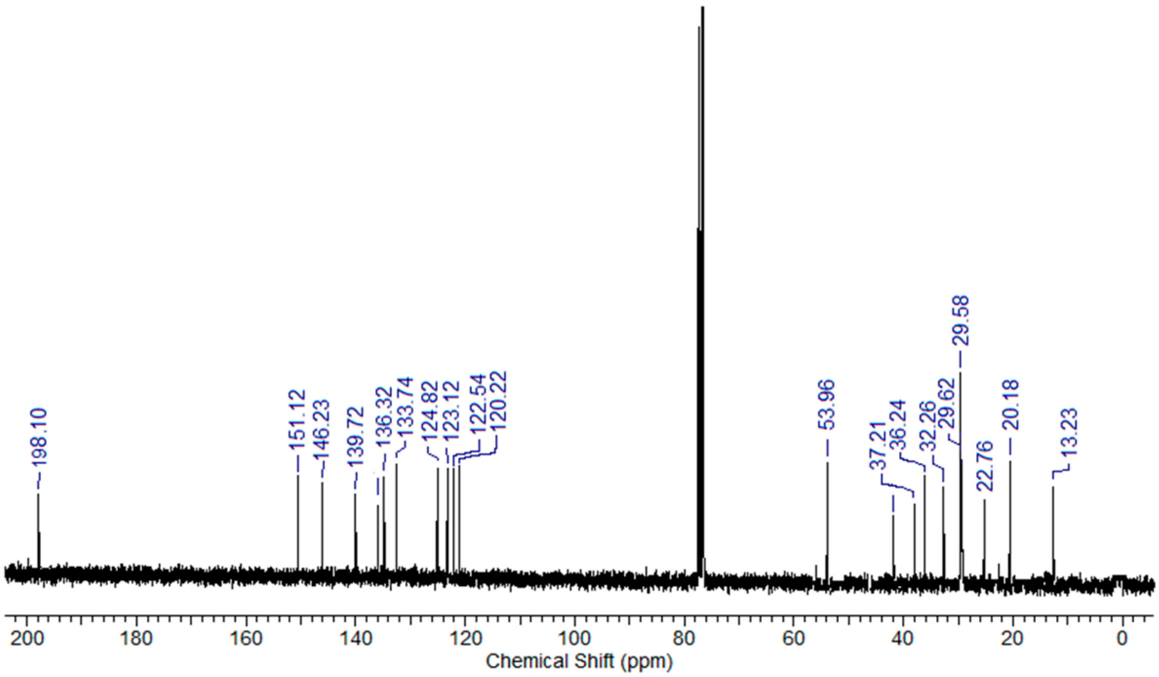

Figure S8. ¹³C NMR spectra of compound **4b**.

^{1}H NMR spectrum ($CDCl_3$) δ ppm: 7.67-7.81 (m, 4H), 7.27 (s, 2H), 4.15 (s, 1H), 3.82 (d, *J* = 10.38 Hz, 1H), 3.63 (d, *J* = 9.77 Hz, 1H), 2.75 (s, 4H), 2.10 (br. s., 1H), 1.64-1.73 (m, 5H), 1.31 (d, *J* = 6.71 Hz, 18H), 0.88 (s, 3H).

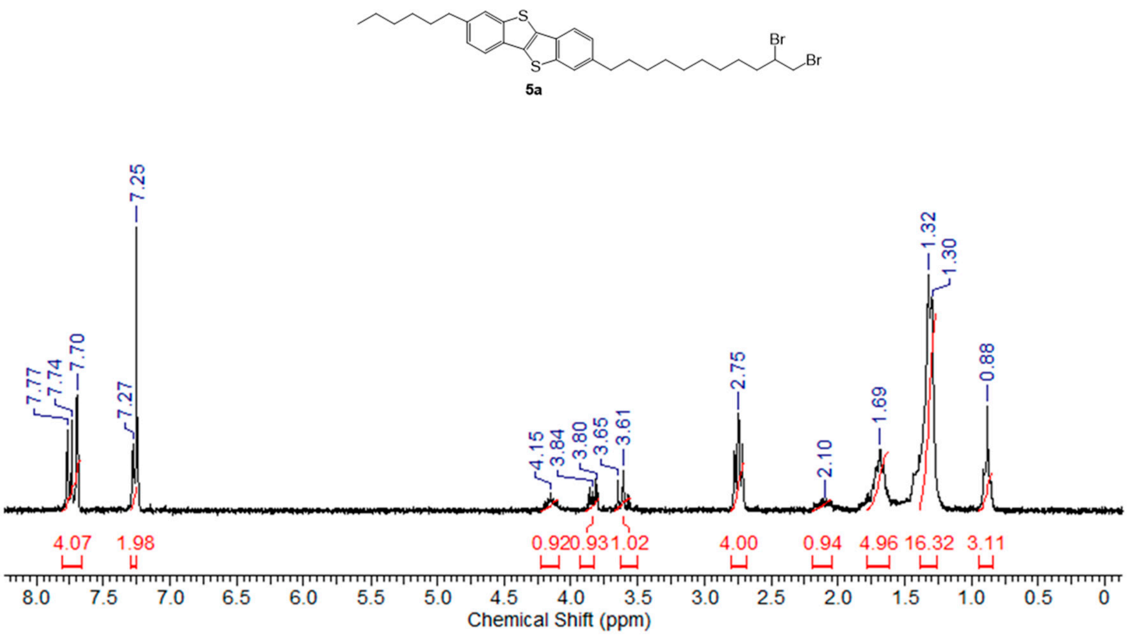

Figure S9. ^{1}H NMR spectra of compound **5a**.

^{1}H NMR spectrum ($CDCl_3$) δ ppm: 7.68-7.81 (m, 4H), 7.23-7.28 (m, 1H), 7.24-7.25 (m, 1H), 4.10-4.24 (m, 1H), 3.81-3.88 (m, 1H), 3.57-3.66 (m, 1H), 2.71-2.82 (m, 4H), 2.14-2.26 (m, 1H), 1.62-1.90 (m, 6H), 1.24-1.38 (m, 10H), 0.83-0.93 (m, 3H).

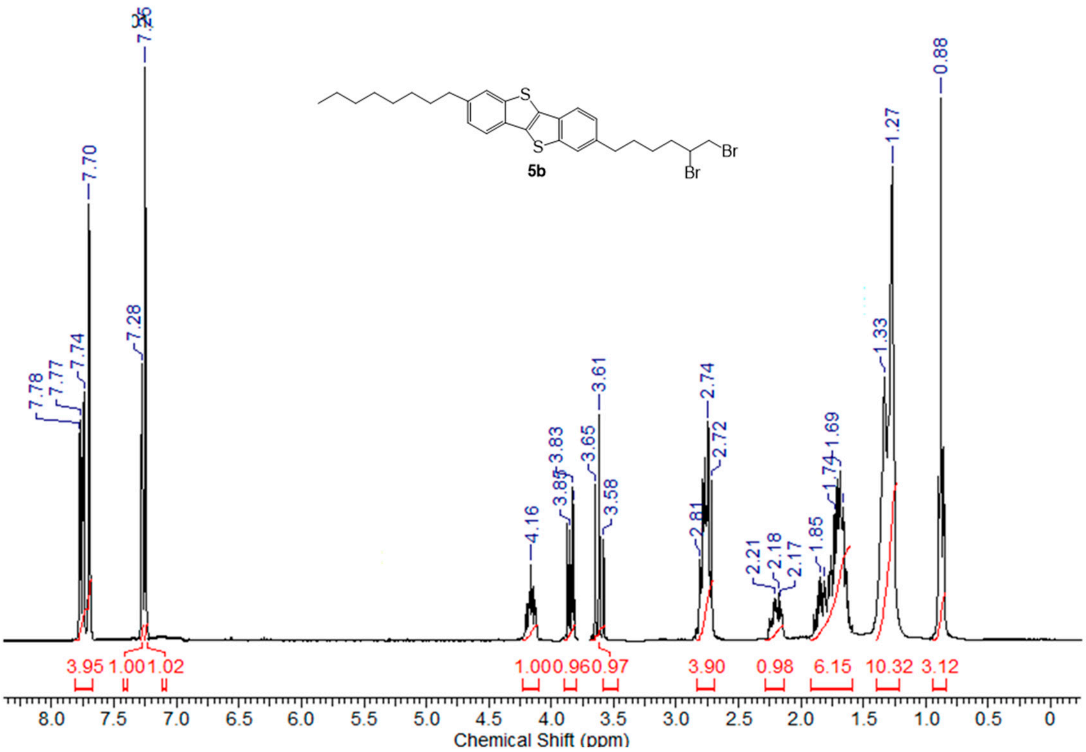

Figure S10. ^{1}H NMR spectra of compound **5b**.

^{13}C NMR spectrum ($CDCl_3$) δ ppm: 142.41, 140.10, 139.17, 125.70, 123.29, 121.15, 52.76, 36.23, 36.10, 35.81, 31.86, 31.69, 30.78, 29.46, 29.24, 26.37, 22.65, 14.10.

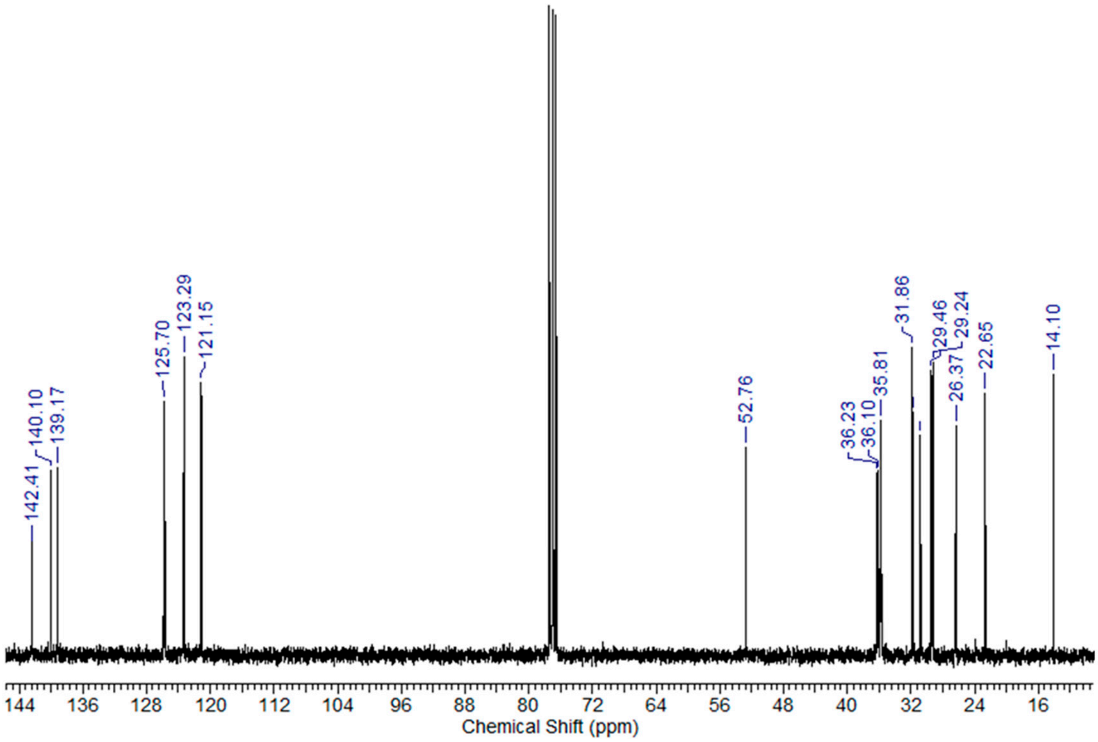

Figure S11. ^{13}C NMR spectra of compound **5b**.

^{1}H NMR spectrum ($CDCl_3$) δ ppm: 7.68-7.81 (m, 4H), 7.24-7.27 (m, 2H), 5.71-5.89 (m, 1H), 4.87-5.06 (m, 2H), 2.74 (t, J = 7.63 Hz, 4H), 1.97-2.09 (m, 2H), 1.61-1.77 (m, 4H), 1.30 (d, J = 13.12 Hz, 18H), 0.83-0.93 (m, 3H).

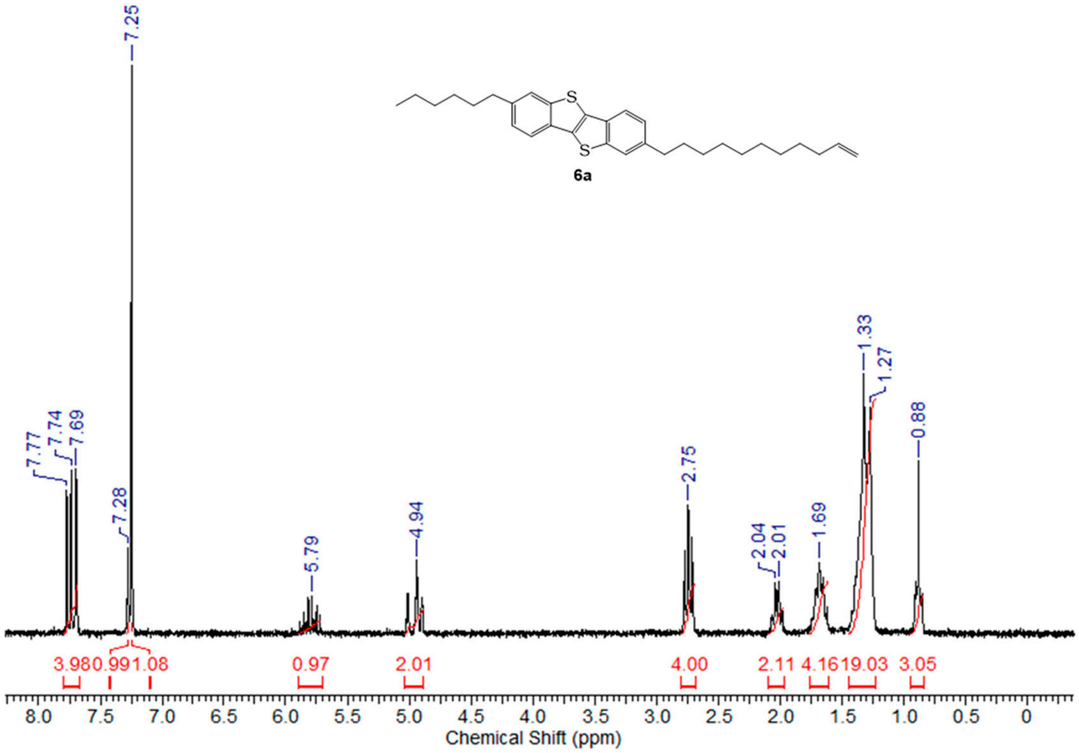

Figure S12. ^{1}H NMR spectra of compound **6a**.

^{1}H NMR spectrum ($CDCl_3$) δ ppm: 7.66-7.81 (m, 4H), 7.22-7.32 (m, 2H), 5.73-5.91 (m, 1H), 4.92-5.09 (m, 2H), 2.70-2.82 (m, 4H), 2.05-2.20 (m, 2H), 1.63-1.81 (m, 4H), 1.22-1.59 (m, 12H), 0.82-0.92 (m, 3H).

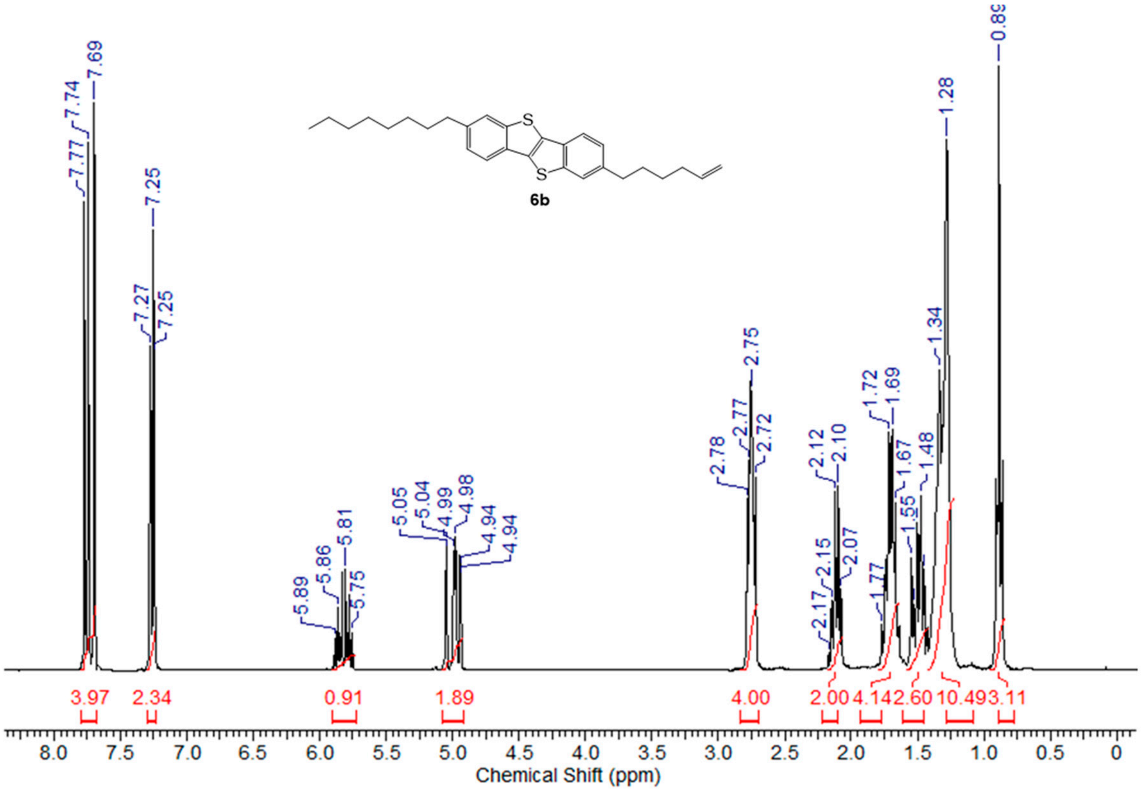

Figure S13. ^{1}H NMR spectra of compound **6b**.

^{13}C NMR spectrum ($CDCl_3$) δ ppm: 142.37, 140.04, 139.72, 138.75, 125.79, 123.29, 121.05, 114.50, 36.10, 33.62, 31.87, 31.10, 29.48, 29.25, 28.50, 22.65, 14.10.

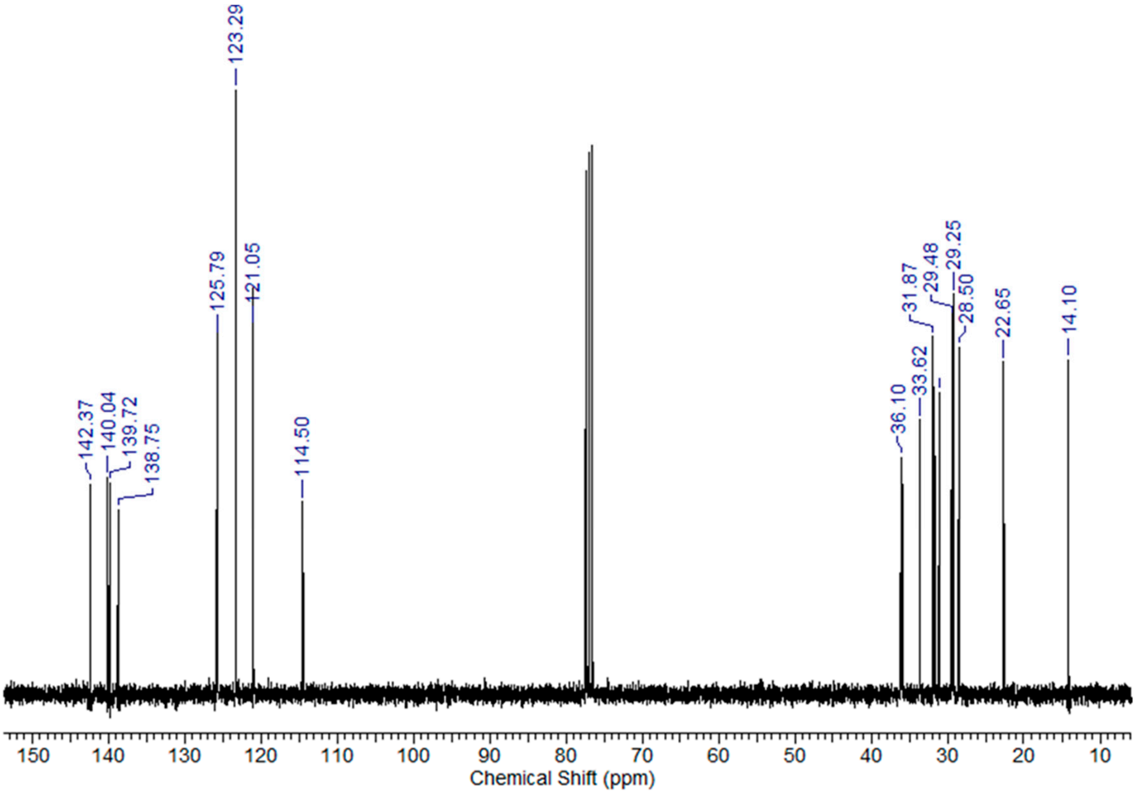

Figure S14. ^{13}C NMR spectra of compound **6b**.

^{1}H NMR spectrum ($CDCl_3$) δ ppm: 7.68-7.82 (m, 4H), 7.26-7.28 (m, 2H), 4.63-4.68 (m, 1H), 2.70-2.78 (m, 4H), 1.64-1.72 (m, 4H), 1.29 (d, *J* = 16.18 Hz, 34 H), 0.83-0.92 (m, 3H), 0.549-0.52 (m, 2H), 0.15 (d, *J* = 2.75 Hz, 6H), 0.04 (s, 6H).

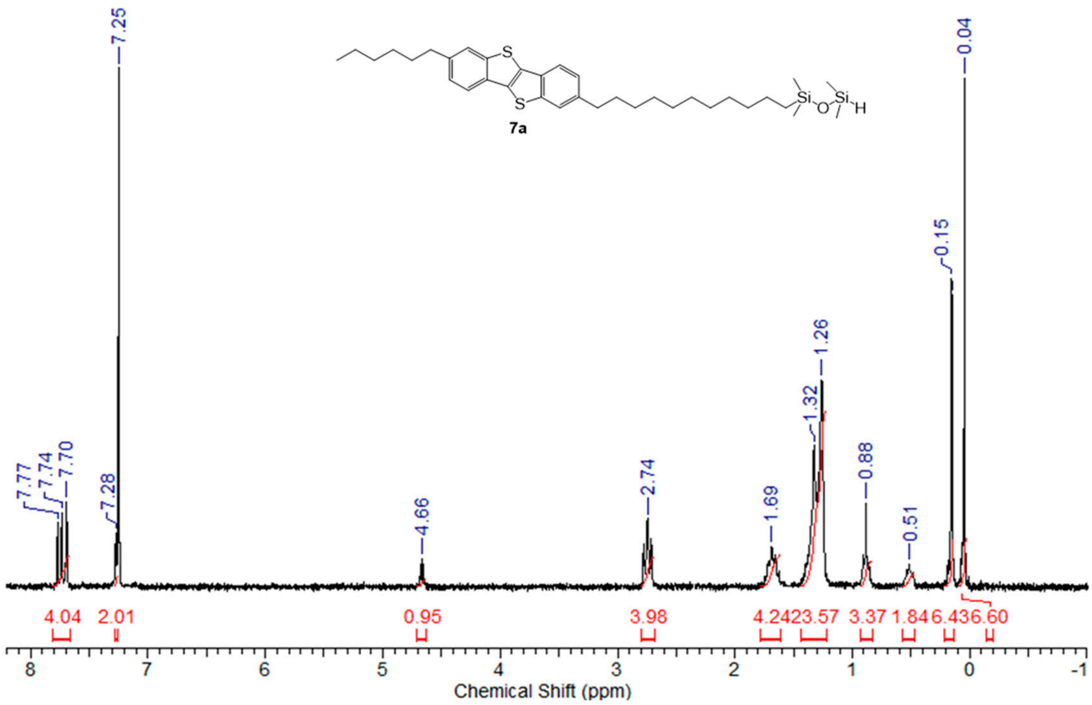

Figure S15. ^{1}H NMR spectra of compound **7a**.

^{1}H NMR spectrum ($CDCl_3$) δ ppm: 7.67-7.80 (m, 4H), 7.25-7.31 (m, 2H), 4.63-4.71 (m, 1H), 2.75 (t, *J* = 7.63 Hz, 4H), 1.66-1.71 (m, 4H), 1.22-1.40 (m, 16H), 0.82-0.93 (m, 3H), 0.54 (d, *J* = 8.85 Hz, 2H), 0.15 (d, *J* = 2.75 Hz, 6H), 0.02-0.09 (m, 6H).

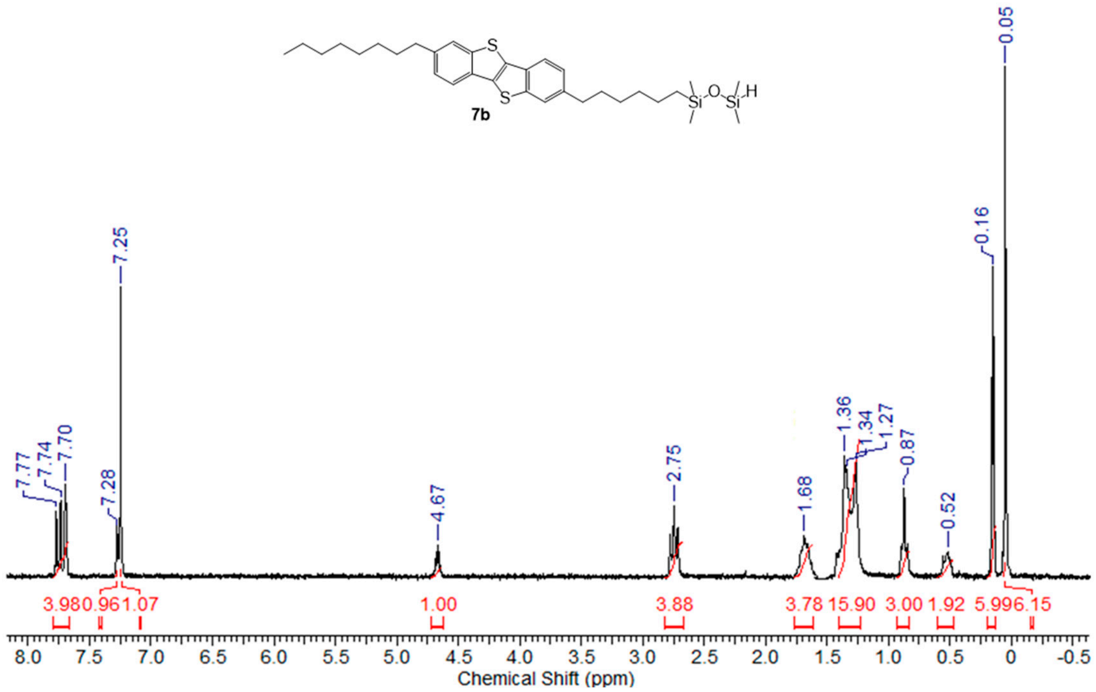

Figure S16. ^{1}H NMR spectra of compound **7b**.

^{13}C NMR spectrum ($CDCl_3$) δ ppm: 142.35, 140.02, 132.49, 125.78, 123.28, 121.02, 36.10, 33.23, 31.87, 31.70, 31.62, 29.47, 29.30, 29.24, 29.00, 23.11, 22.64, 18.11, 14.09, 0.90, 0.03.

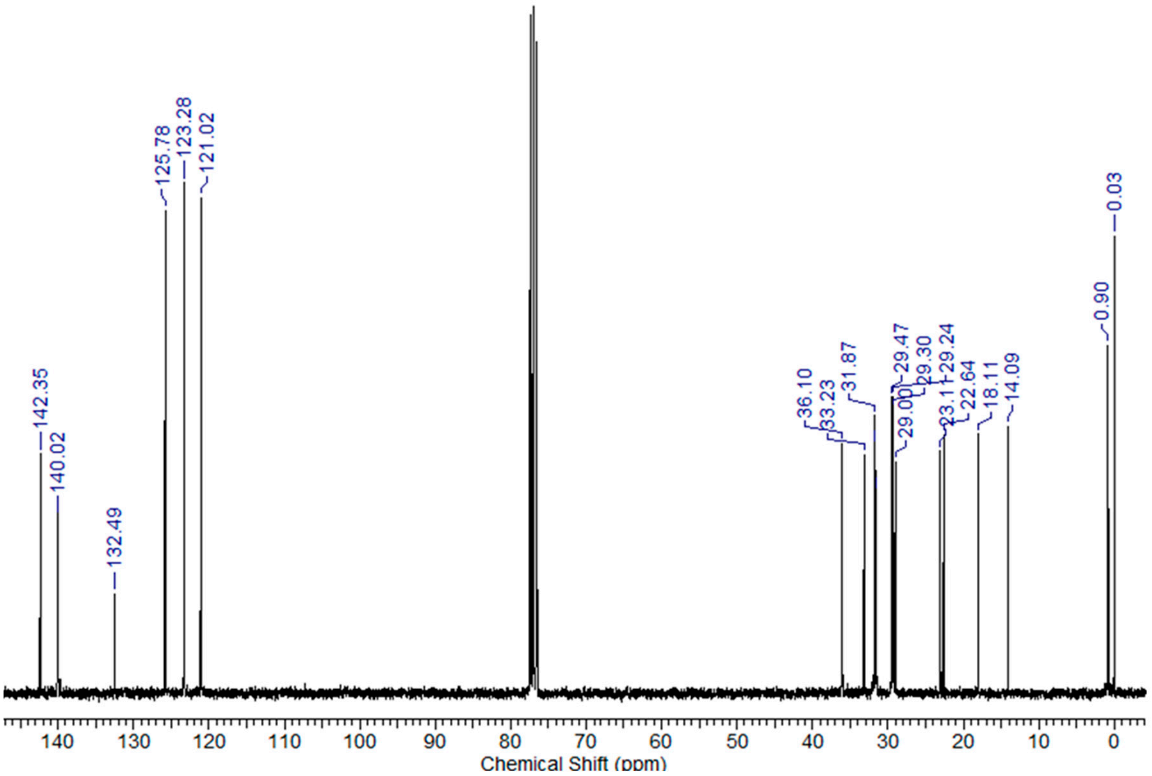

Figure S17. ^{13}C NMR spectra of compound **7b**.

¹H NMR spectrum (CDCl₃) δ ppm: 7.64-7.79 (m, 16H), 7.21-7.30 (m, 8H), 2.68-2.81 (m, 16H), 1.67 (d, J = 7.15 Hz, 16H), 1.30 (d, J = 19.07 Hz, 96H), 0.84-0.95 (m, 12H), 0.46-0.66 (m, 24H), 0.04 (s, 48H).

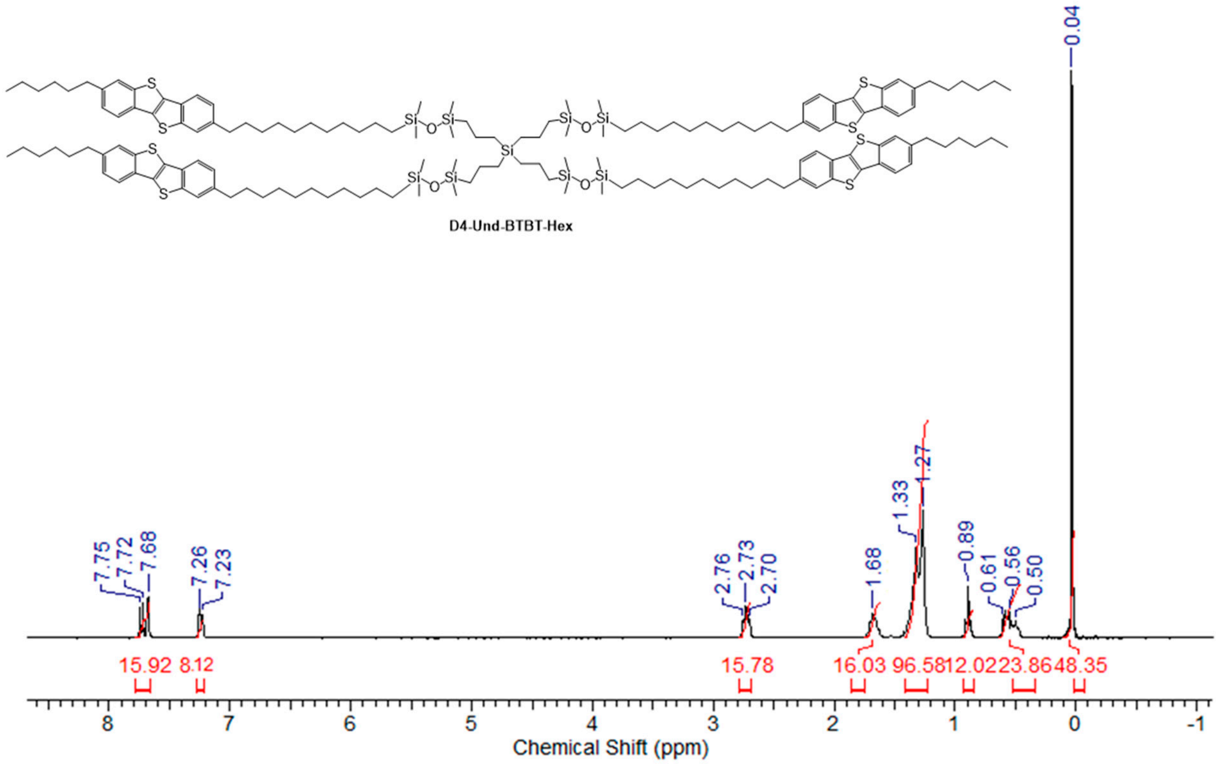

Figure S18. ¹H NMR spectra of compound **D4-Und-BTBT-Hex**.

¹³C NMR spectrum (CDCl₃) δ ppm: 142.35, 140.00, 132.49, 131.14, 125.77, 123.26, 121.01, 36.10, 33.51, 31.72, 31.67, 29.75, 29.66, 29.57, 29.45, 29.37, 28.98, 23.42, 23.32, 22.60, 18.43, 17.98, 17.19, 14.11, 0.51, 0.43.

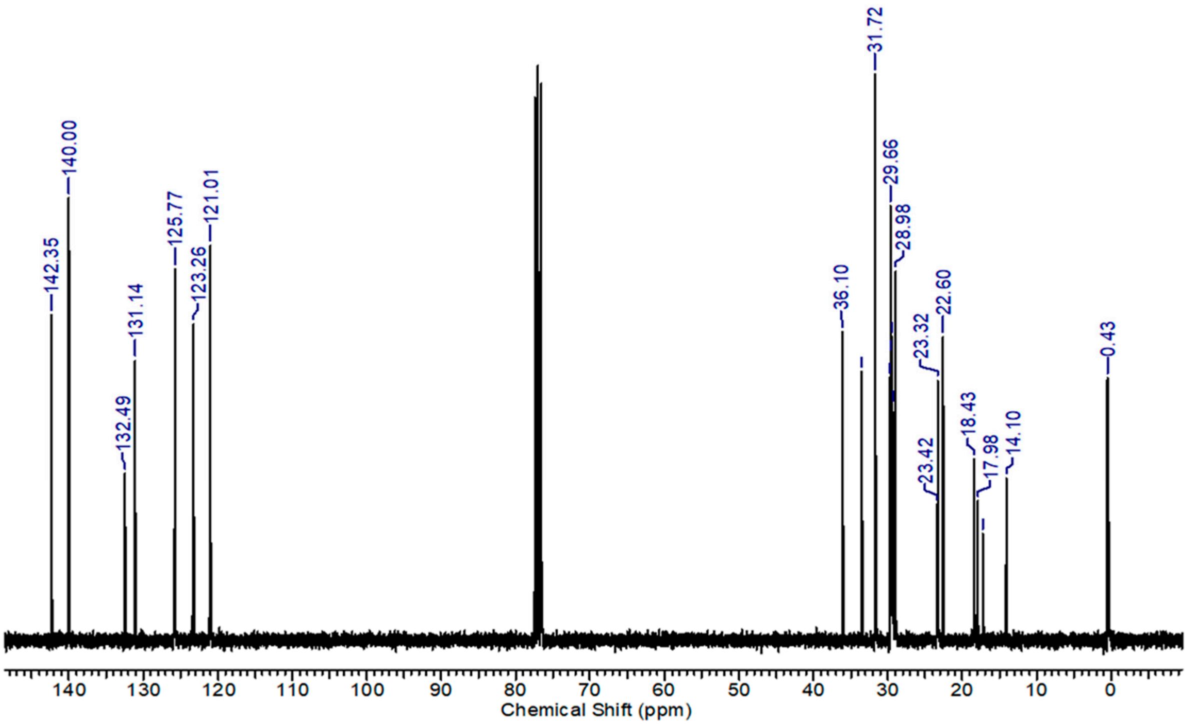

Figure S19. ¹³C NMR spectra of compound **D4-Und-BTBT-Hex**.

²⁹Si NMR spectrum (CDCl₃) δ ppm: 7.30, 6.72.

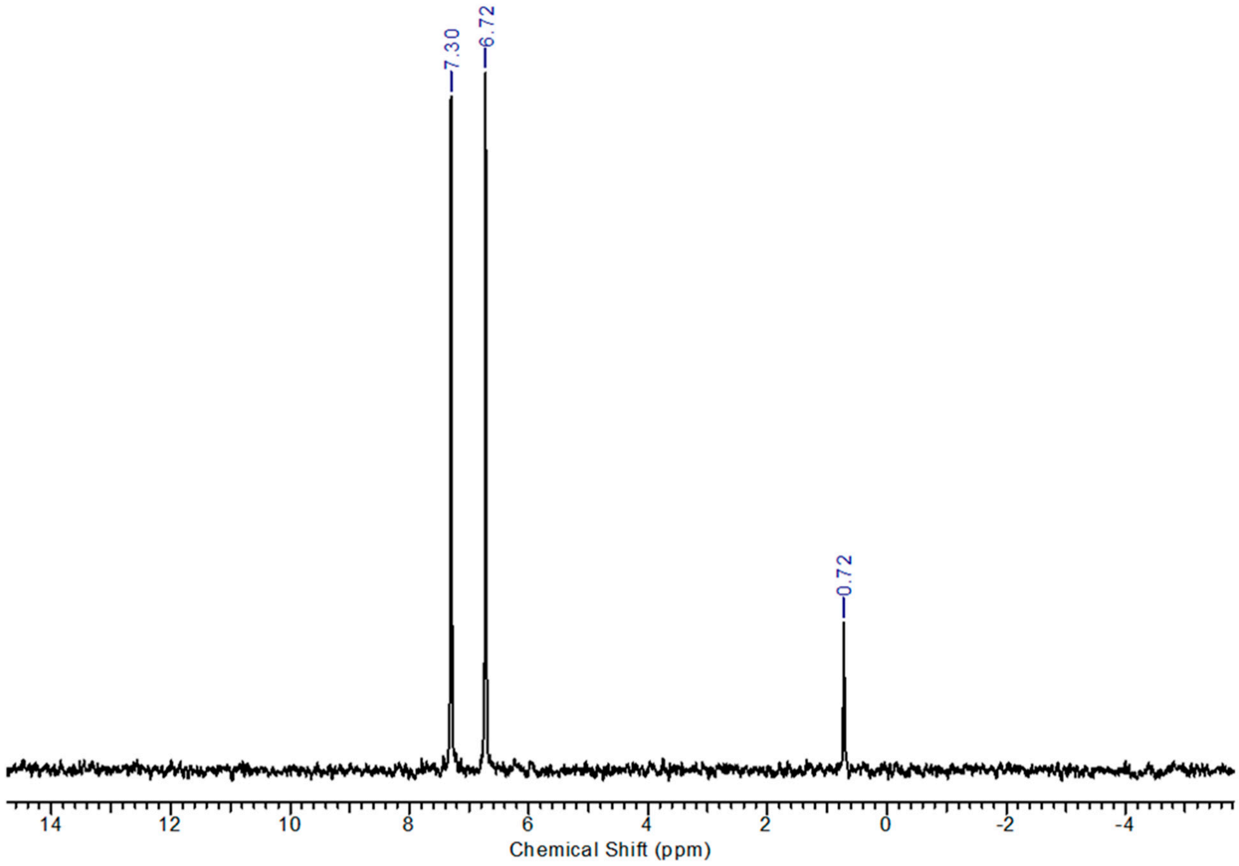

Figure S20. ^{29}Si NMR spectra of compound **D4-Und-BTBT-Hex**.

^{1}H NMR spectrum ($CDCl_3$) δ ppm: 7.63-7.76 (m, 16H), 7.22 (m, 8H), 2.72 (m, 16H), 1.61-1.70 (m, 16H), 1.30 (d, *J* = 17.70 Hz, 72H), 0.83-0.93 (m, 12H), 0.44-0.63 (m, 24H), 0.02-0.08 (m, 48H).

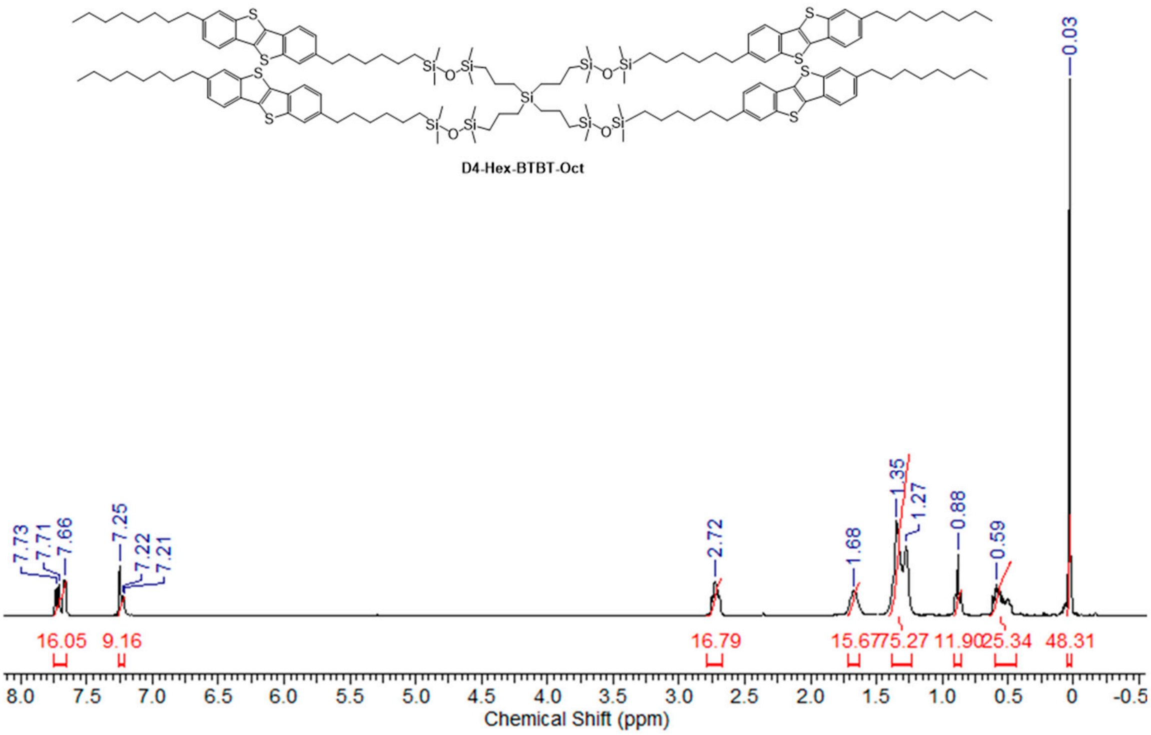

Figure S21. ^{1}H NMR spectra of compound **D4-Hex-BTBT-Oct**.

^{13}C NMR spectrum ($CDCl_3$) δ ppm: 142.34, 139.99, 131.12, 125.75, 123.25, 120.99, 36.09, 33.32, 31.87, 31.69, 29.47, 29.32, 29.24, 29.1123.42, 23.46, 22.65, 18.43, 17.98, 17.19, 14.10, 0.42.

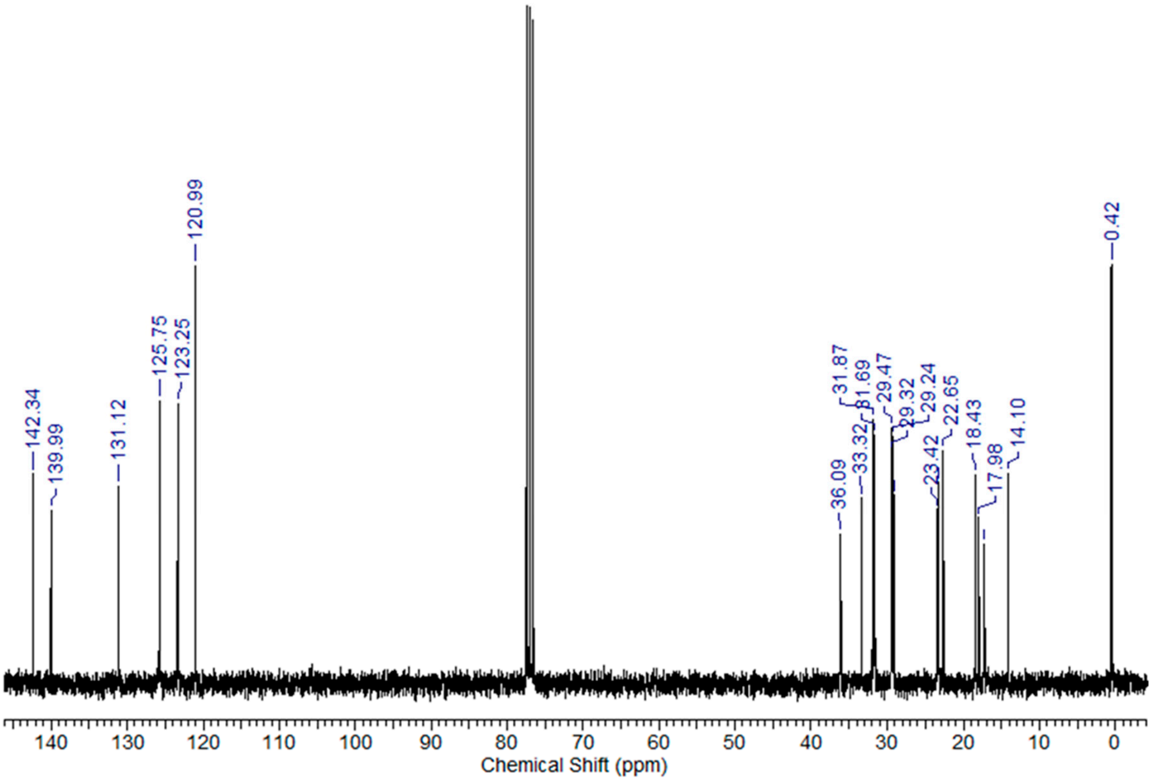

Figure S22. ^{13}C NMR spectra of compound **D4-Hex-BTBT-Oct**.

^{29}Si NMR spectrum ($CDCl_3$) δ ppm: 7.31, 6.72.

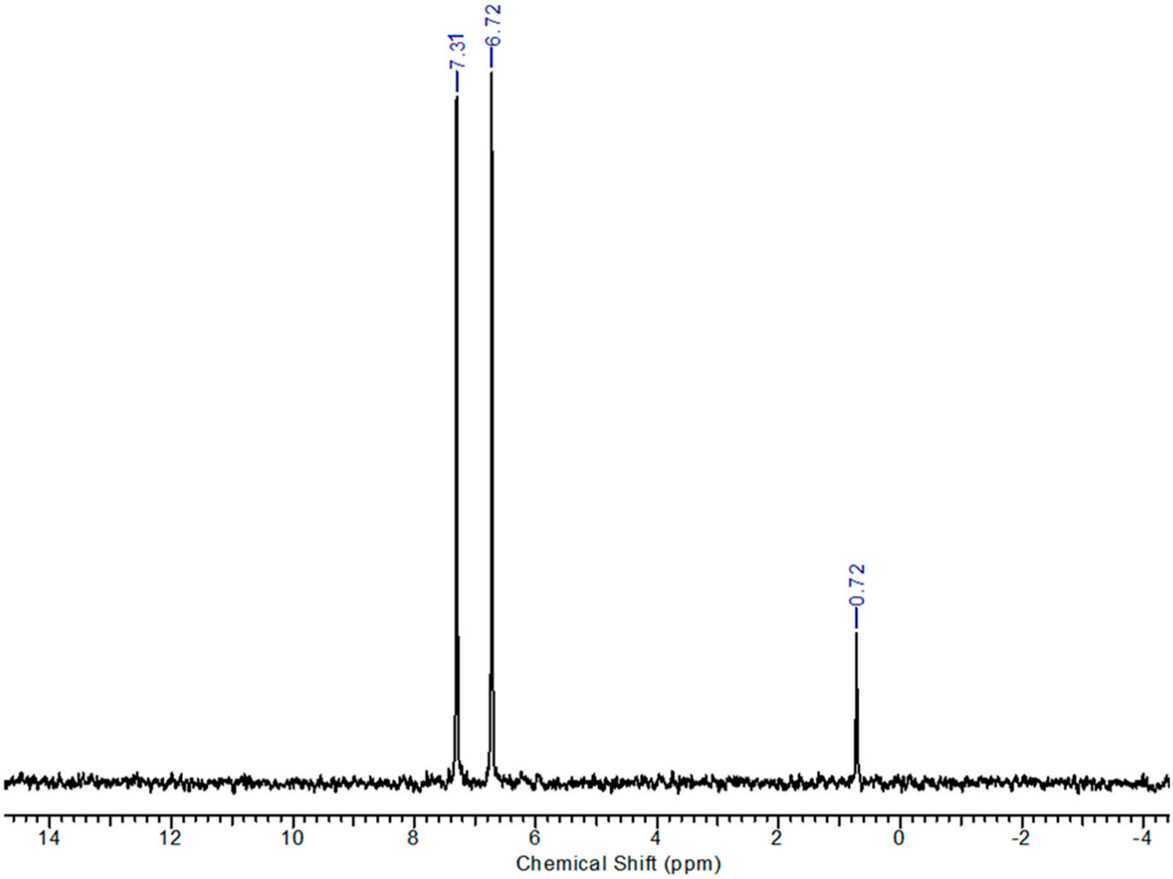

Figure S23. ^{29}Si NMR spectra of compound **D4-Hex-BTBT-Oct**.

2. GPC curves

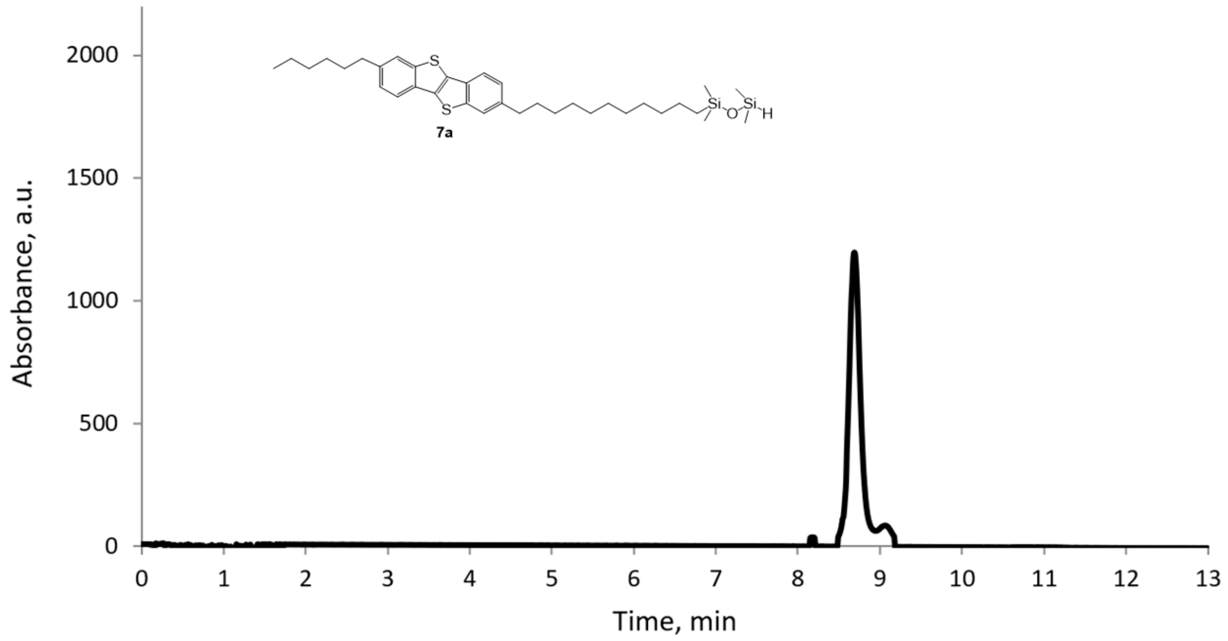

Figure S24. GPC curve of compound **7a**.

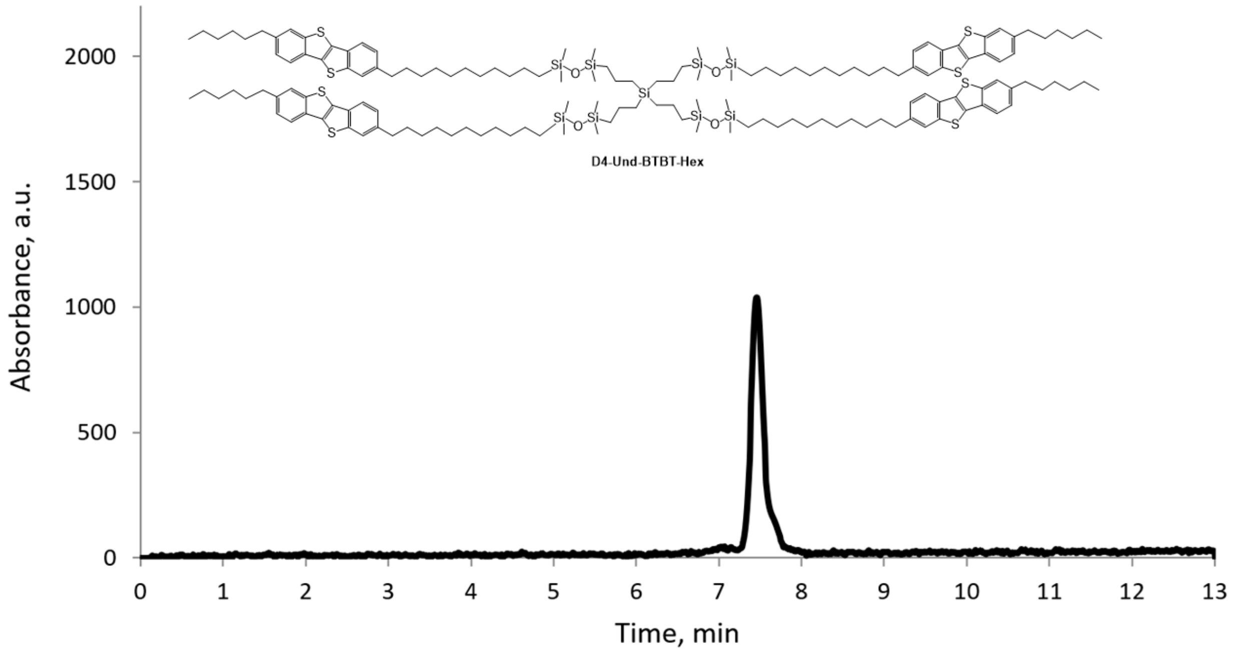

Figure S25. GPC curve of compound **D4-Und-BTBT-Hex**.

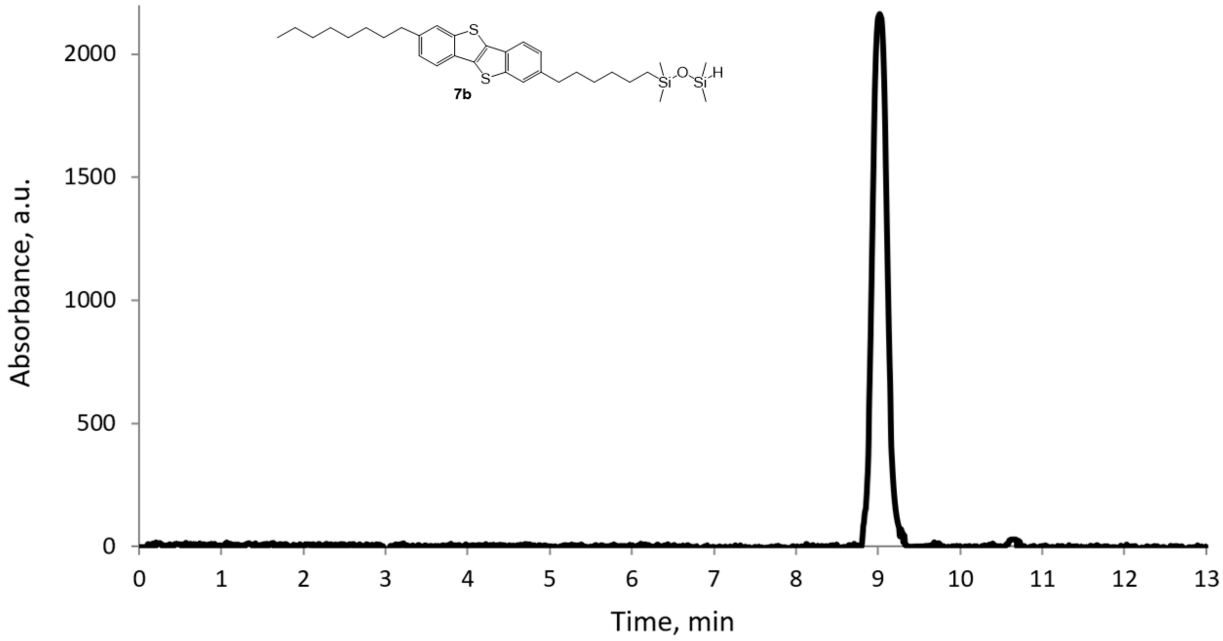

Figure S26. GPC curve of compound **7b**.

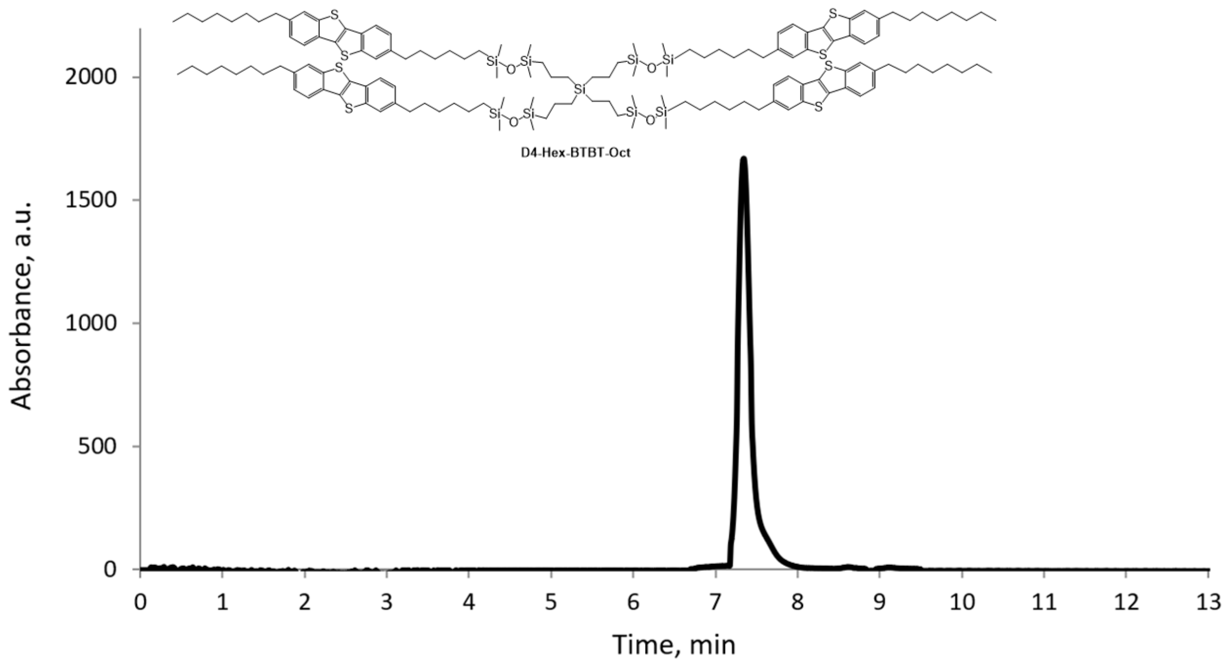

Figure S27. GPC curve of compound **D4-Hex-BTBT-Oct**.

3. MALDI spectra

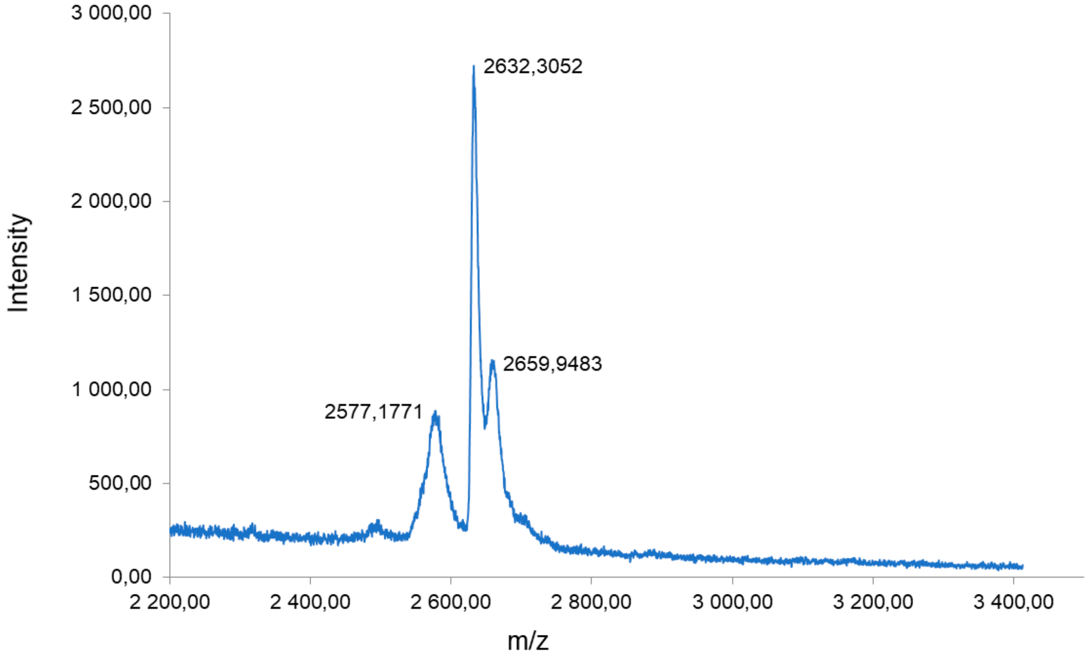

Figure S28. Mass spectrum of the compound **D4-Und-BTBT-Hex**.

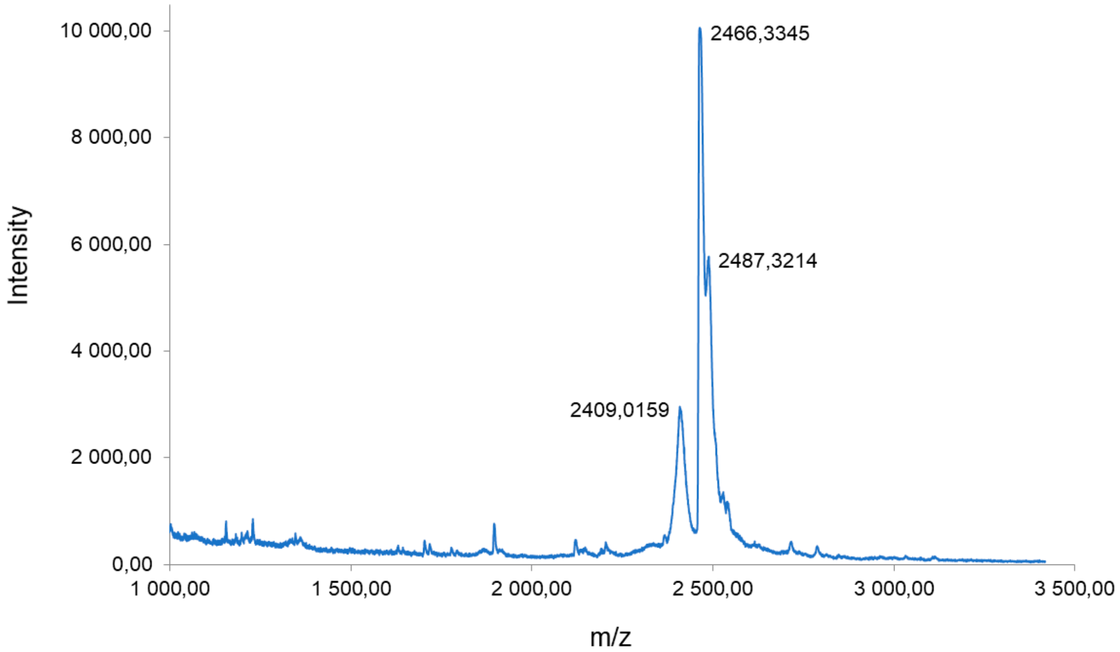

Figure S29. Mass spectrum of the compound **D4-Hex-BTBT-Oct**.

4. DSC data

Table S1. Temperatures and enthalpies of phase transitions

Sample	Heating	T_m, °C	ΔH_m, J g^{-1}	T_i, °C	ΔH_i, J g^{-1}
**D4-Und-BTBT-Hex**	1st	73,80	23	134	10
	2nd	77	22	127	11
**D4-Hex-BTBT-Oct**	1st	31	16	118	12
	2nd	-	-	118	12

5. X-ray diffraction

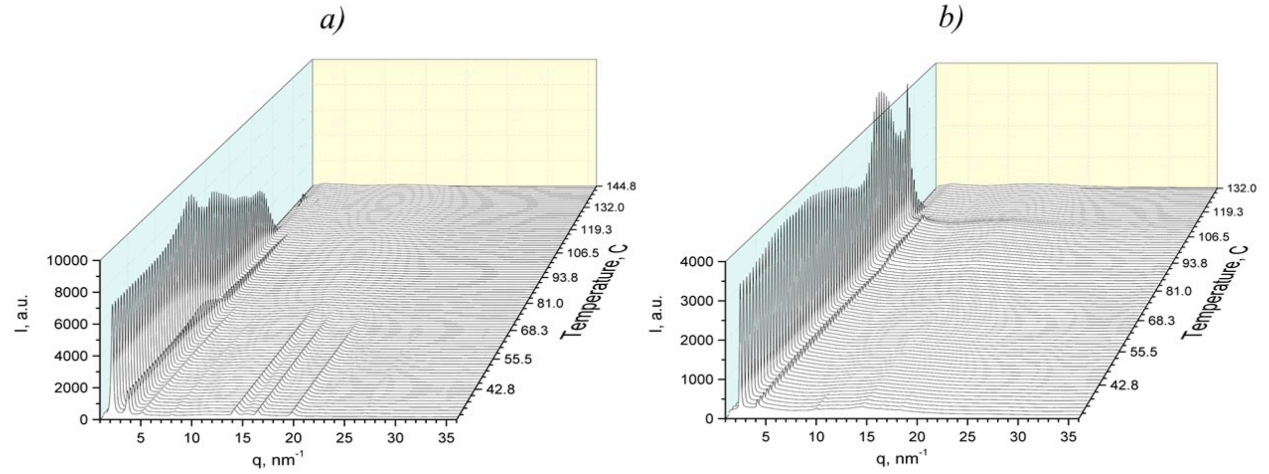

Figure S30. Diffraction patterns during heating in situ in an X-ray beam at a rate of 6 °C/min: *a)* sample **D4-Und-BTBT-Hex**; *b)* sample **D4-Hex-BTBT-Oct**.

6. Electrical performance data for obtained OFETs

Table S2. Original data for electrical characteristics of the devices (batch 2).

	D4-Und-BTBT-Hex			
**Device**	**6**		**17**	
**C, g L^{-1}**	**1.0**		**2.0**	
**SGVolts**	**SDAmpers**	**Sqrt(I)**	**SDAmpers**	**Sqrt(I)**
**Volts**	**Ampers**	**sqrt(A)**	**Ampers**	**sqrt(A)**
20	-4,04E-10	2,01E-05	-1,97E-11	4,44E-06
18,5	-2,58E-10	1,61E-05	-1,37E-11	3,71E-06
17	-2,49E-10	1,58E-05	-1,19E-11	3,45E-06
15,5	-2,24E-10	1,50E-05	-1,22E-11	3,50E-06
14	-2,26E-10	1,50E-05	-1,22E-11	3,49E-06
12,5	-1,96E-10	1,40E-05	-1,26E-11	3,55E-06
11	-1,99E-10	1,41E-05	-1,21E-11	3,47E-06
9,5	-1,89E-10	1,37E-05	-1,25E-11	3,53E-06
8,0	-1,49E-10	1,22E-05	-1,18E-11	3,43E-06
6,5	-1,19E-10	1,09E-05	-1,15E-11	3,40E-06
5	-9,49E-11	9,74E-06	-1,24E-11	3,52E-06
3,5	-7,28E-11	8,53E-06	-1,19E-11	3,45E-06
2	-6,19E-11	7,87E-06	-1,24E-11	3,51E-06
0,5	-4,84E-11	6,95E-06	-1,15E-11	3,39E-06
-1	-6,89E-10	2,62E-05	-3,71E-10	1,93E-05
-2,5	-4,12E-09	6,42E-05	-2,79E-09	5,28E-05
-4	-1,23E-08	1,11E-04	-8,93E-09	9,45E-05
-5,5	-2,85E-08	1,69E-04	-2,02E-08	1,42E-04
-7	-5,54E-08	2,35E-04	-4,16E-08	2,04E-04
-8,5	-9,74E-08	3,12E-04	-7,99E-08	2,83E-04
-10	-1,64E-07	4,04E-04	-1,43E-07	3,78E-04
-11,5	-2,52E-07	5,02E-04	-2,40E-07	4,89E-04
-13	-3,75E-07	6,12E-04	-3,77E-07	6,14E-04
-14,5	-5,32E-07	7,29E-04	-5,63E-07	7,50E-04
-16	-7,35E-07	8,57E-04	-7,93E-07	8,90E-04
-17,5	-9,73E-07	9,86E-04	-1,05E-06	1,03E-03
-19	-1,26E-06	1,12E-03	-1,38E-06	1,17E-03
-20,5	-1,59E-06	1,26E-03	-1,75E-06	1,32E-03
-22	-1,98E-06	1,41E-03	-2,19E-06	1,48E-03
-23,5	-2,42E-06	1,56E-03	-2,62E-06	1,62E-03
-25	-2,92E-06	1,71E-03	-3,15E-06	1,77E-03
-26,5	-3,46E-06	1,86E-03	-3,69E-06	1,92E-03
-28	-4,06E-06	2,01E-03	-4,35E-06	2,08E-03
-29,5	-4,72E-06	2,17E-03	-4,97E-06	2,23E-03
-31	-5,38E-06	2,32E-03	-5,67E-06	2,38E-03
-32,5	-6,12E-06	2,47E-03	-6,43E-06	2,54E-03

-34	-6,93E-06	2,63E-03	-7,17E-06	2,68E-03
-35,5	-7,75E-06	2,78E-03	-8,02E-06	2,83E-03
-37	-8,56E-06	2,93E-03	-8,87E-06	2,98E-03
-38,5	-9,41E-06	3,07E-03	-9,71E-06	3,12E-03
-40	-1,02E-05	3,20E-03	-1,06E-05	3,26E-03
-38,5	-7,76E-06	2,79E-03	-8,11E-06	2,85E-03
-37	-6,00E-06	2,45E-03	-6,57E-06	2,56E-03
-35,5	-4,63E-06	2,15E-03	-5,35E-06	2,31E-03
-34	-3,50E-06	1,87E-03	-4,36E-06	2,09E-03
-32,5	-2,62E-06	1,62E-03	-3,46E-06	1,86E-03
-31	-1,90E-06	1,38E-03	-2,71E-06	1,65E-03
-29,5	-1,35E-06	1,16E-03	-2,07E-06	1,44E-03
-28	-9,24E-07	9,61E-04	-1,51E-06	1,23E-03
-26,5	-6,09E-07	7,80E-04	-1,05E-06	1,02E-03
-25	-3,84E-07	6,20E-04	-6,91E-07	8,31E-04
-23,5	-2,31E-07	4,81E-04	-4,25E-07	6,52E-04
-22	-1,50E-07	3,87E-04	-2,43E-07	4,93E-04
-20,5	-7,78E-08	2,79E-04	-1,28E-07	3,58E-04
-19	-5,33E-08	2,31E-04	-6,73E-08	2,59E-04
-17,5	-2,27E-08	1,51E-04	-3,49E-08	1,87E-04
-16	-1,26E-08	1,12E-04	-1,72E-08	1,31E-04
-14,5	-6,69E-09	8,18E-05	-7,33E-09	8,56E-05
-13	-4,08E-09	6,39E-05	-3,22E-09	5,67E-05
-11,5	-2,11E-09	4,59E-05	-1,42E-09	3,77E-05
-10	-1,34E-09	3,66E-05	-6,86E-10	2,62E-05
-8,5	-8,59E-10	2,93E-05	-3,12E-10	1,77E-05
-7	-5,72E-10	2,39E-05	-2,19E-10	1,48E-05
-5,5	-4,05E-10	2,01E-05	-1,76E-10	1,32E-05
-4	-3,16E-10	1,78E-05	-1,53E-10	1,24E-05
-2,5	-2,76E-10	1,66E-05	-1,40E-10	1,18E-05
-1	-3,15E-10	1,78E-05	-1,31E-10	1,14E-05
0,5	-2,40E-10	1,55E-05	-1,23E-10	1,11E-05
2	-2,35E-10	1,53E-05	-1,21E-10	1,10E-05
3,5	-3,59E-10	1,89E-05	-1,19E-10	1,09E-05
5	-2,48E-10	1,58E-05	-1,12E-10	1,06E-05
6,5	-2,55E-10	1,60E-05	-1,10E-10	1,05E-05
8,0	-2,68E-10	1,64E-05	-1,10E-10	1,05E-05
9,5	-2,86E-10	1,69E-05	-1,08E-10	1,04E-05
11	-4,85E-10	2,20E-05	-1,07E-10	1,03E-05
12,5	-3,84E-10	1,96E-05	-1,06E-10	1,03E-05
14	-3,51E-10	1,87E-05	-1,06E-10	1,03E-05
15,5	-7,25E-10	2,69E-05	-1,05E-10	1,02E-05
17	-3,03E-09	5,51E-05	-1,04E-10	1,02E-05
18,5	-7,12E-10	2,67E-05	-1,04E-10	1,02E-05

20	-6,39E-10	2,53E-05	-1,04E-10	1,02E-05

Table S3. Electrical performance dataset for BTBT-based OFETs fabricated under different conditions applied by spin-coating method.

Organic semiconductor	C, g L^{-1}	μ_{max} (μ_{ave}), $cm^2 \cdot V^{-1} \cdot S^{-1}$	V_{th}, V	$I_{on/off}$	Substrate treatment
**D4-Und-BTBT-Hex** (batch 1)	1.0	1.6×10^{-3} (5.2×10^{-4})	−5.3 to 4.9	$10^5 - 10^7$	plasma
	2.0	1.8×10^{-3} (8.0×10^{-4})	−5.8 to 13.1	$10^4 - 10^7$	plasma
**D4-Und-BTBT-Hex** (batch 2)	1.0	3.3×10^{-2} (2.0×10^{-2})	−20.1 to −4.0	$10^3 - 10^5$	plasma
	2.0	3.5×10^{-2} (2.1×10^{-2})	−17.1 to −7.0	$10^3 - 10^5$	plasma
**D4-Hex-BTBT-Oct** (batch 1)	1.0	-	-	-	plasma
	2.0	-	-	-	plasma
**D4-Hex-BTBT-Oct** (batch 2)	1.0	3.8×10^{-6} (2.6×10^{-6})	-11 to +14	$10^0 - 10^1$	plasma
	2.0	4.5×10^{-6} (3.4×10^{-6})	-11 to +2	$10^0 - 10^1$	plasma
**D2-Und-BTBT-Hex**[1]	1.0	4.0×10^{-3} (1.0×10^{-3})	-2 to +2	$10^2 - 10^3$	ODMS
	1.3	4.0×10^{-2} (9.0×10^{-3})	-2 to +2	$10^4 - 10^5$	ODMS
	1.4	5.0×10^{-3} (1.0×10^{-3})	-10 to -5	$10^3 - 10^4$	ODMS
	2.0	9.0×10^{-4} (7.0×10^{-4})	-20 to -15	$10^2 - 10^3$	ODMS
**D2-Hex-BTBT-Hex**[2]	1.0	8.0×10^{-3} (3.0×10^{-3})	−3…+3	10^3–10^4	ODMS
**D2-Hex-BTBT-Hex**	2.0	4.7×10^{-2} (2.2×10^{-2})	−14…−10	10^4–10^5	PMMA
**D2-Hept-BTBT-Hex**	1.0	9.0×10^{-3} (4.0×10^{-3})	−3…+2	10^3–10^4	ODMS
**D2-Hept-BTBT-Hex**	2.0	1.6×10^{-1} (2.6×10^{-2})	−9…−3	10^5–10^6	PMMA
**D2-Und-BTBT-Hex**	1.0	4.0×10^{-2} (0.9×10^{-2})	−2…+2	10^4–10^5	ODMS
**D2-Und-BTBT-Hex**	2.0	2.4×10^{-1} (1.0×10^{-1})	−14…−10	10^5–10^6	PMMA

[1] Trul, A.A.; Sizov, A.S.; Chekusova, V.P.; Borshchev, O.V.; Agina, E.V.; Shcherbina, M.A. Bakirov, A.V.; Chvalun, S.N.; Ponomarenko, S.A. Organosilicon dimer of BTBT as a perspective semiconductor material for toxic gas detection with monolayer organic field-effect transistors. *J. Mater. Chem. C*, **2018**, 6, 9649-9659. https://doi.org/10.1039/C8TC02447B

[2] Trul, A.A.; Chekusova, V.P.; Anisimov, D.S.; Borshchev, O.V.; Polinskaya, M.S.; Agina, E.V.; Ponomarenko, S.A. Operationally Stable Ultrathin Organic Field Effect Transistors Based on Siloxane Dimers of Benzothieno[3,2-b][1]Benzothiophene Suitable for Ethanethiol Detection. *Adv. Electron. Mater.* **2022**, 8, 2101039. https://doi.org/10.1002/aelm.202101039

Notes: ODMS – octyldimethylchlorosilane, PMMS – poly(methylmethacrylate).

7. Micrographs of the OFETs surface

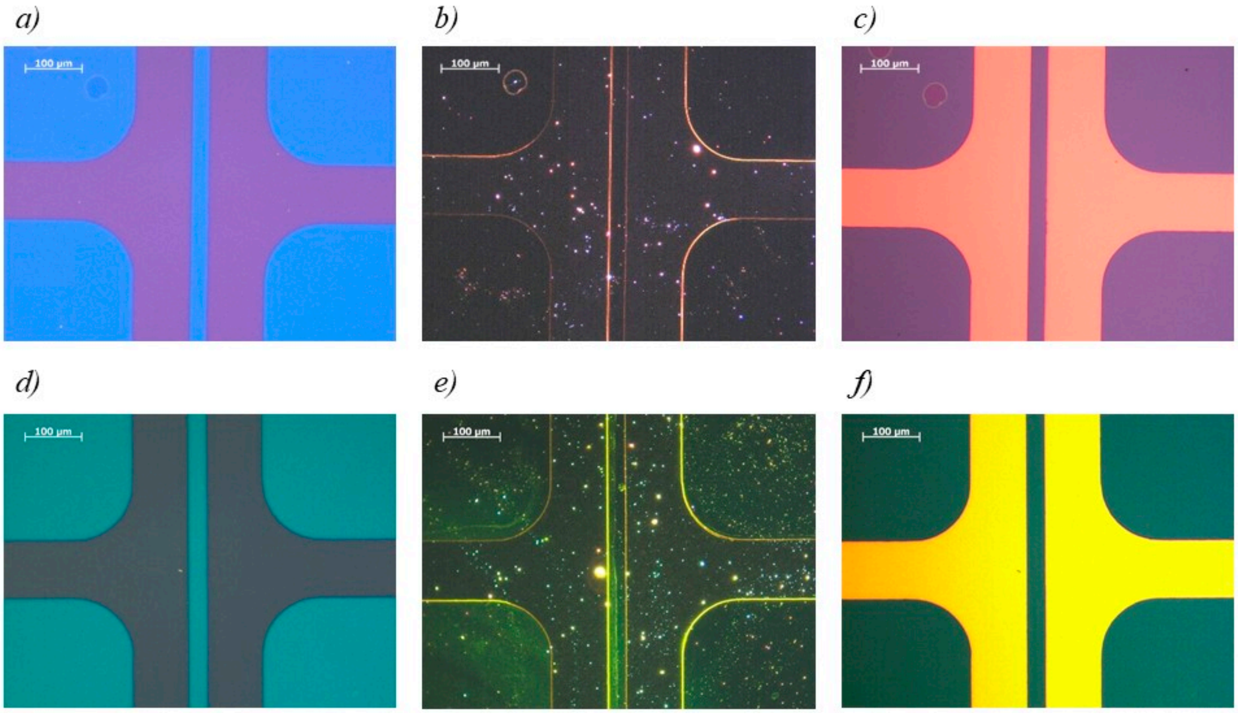

Figure S31. Optical micrographs of **D4-Und-BTBT-Hex** thin films obtained from the solutions with a concentration of 1.0 g L^{-1} (*a*-*c*) and with a concentration of 2.0 g L^{-1} (*d*-*f*) in cross polarizers (*a*, *d*), in dark field (*b*, *e*) and in bright field (*c*, *f*); batch 2.

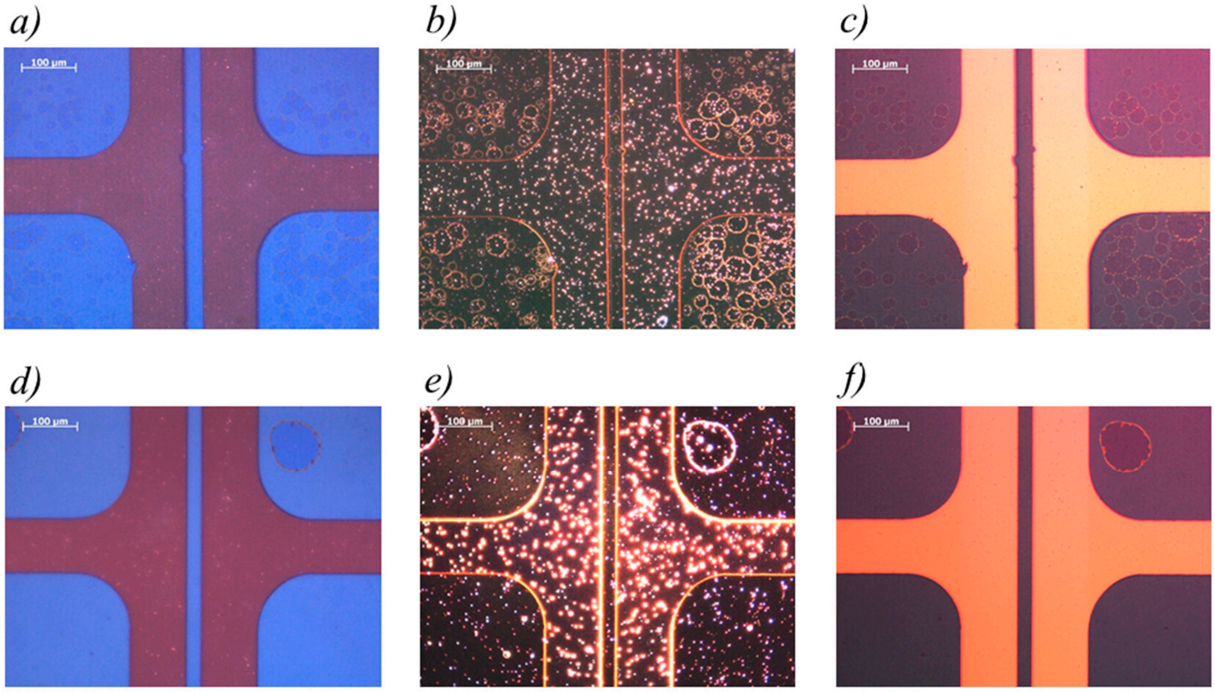

Figure S32. Optical micrographs of **D4-Und-BTBT-Hex** thin films obtained from the solutions with a concentration of 1.0 g L^{-1} (*a*-*c*) and with a concentration of 2.0 g L^{-1} (*d*-*f*) in cross polarizers (*a*, *d*), in dark field (*b*, *e*) and in bright field (*c*, *f*); batch 1.

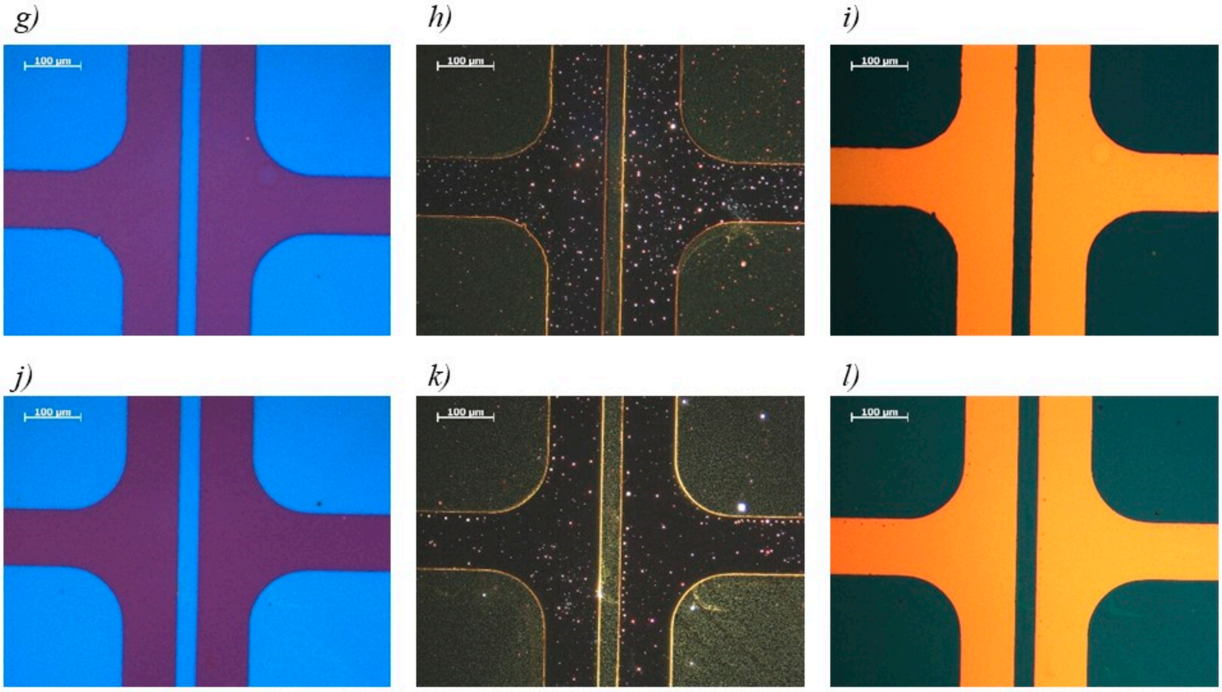

Figure S33. Optical micrographs of **D4-Hex-BTBT-Oct** thin films obtained from the solutions with a concentration of 1.0 g L^{-1} (*g*-*i*) and with a concentration of 2.0 g L^{-1} (*j*-*l*) in cross polarizers (*g*, *j*), in dark field (*h*, *k*) and in bright field (*i*, *l*); batch 2.

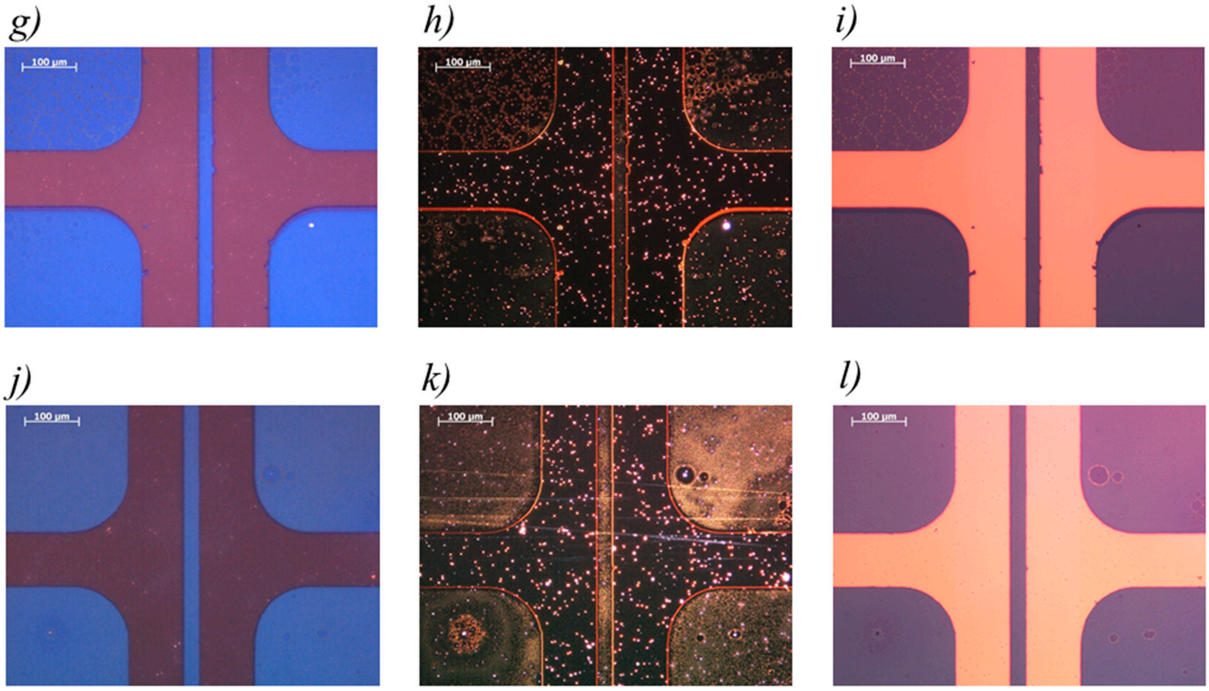

Figure S34. Optical micrographs of **D4-Hex-BTBT-Oct** thin films obtained from the solutions with a concentration of 1.0 g L^{-1} (*g*-*i*) and with a concentration of 2.0 g L^{-1} (*j*-*l*) in cross polarizers (*g*, *j*), in dark field (*h*, *k*) and in bright field (*i*, *l*); batch 1.

8. AFM images of the OFETs surface

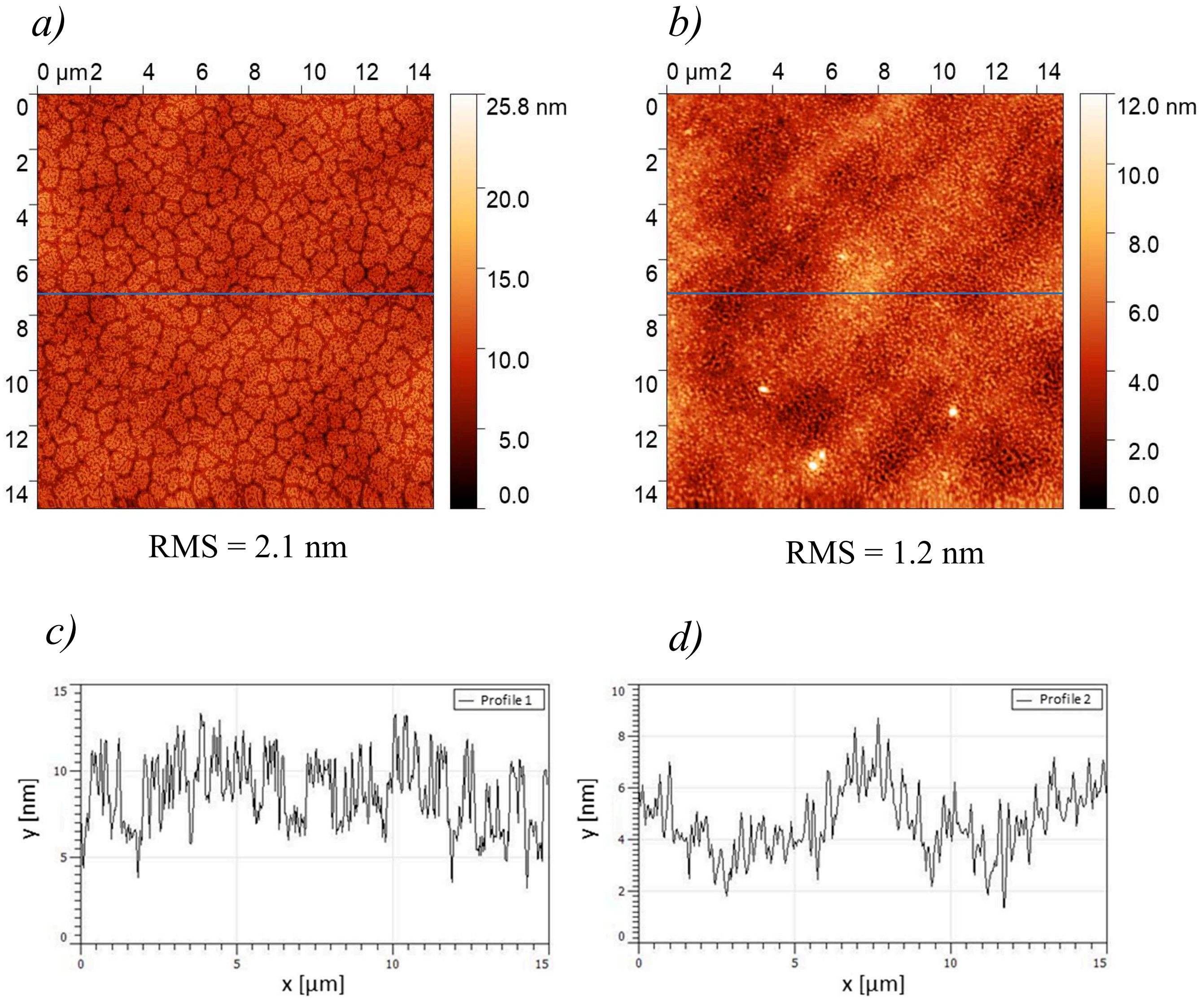

Figure S35. AFM topography images of **D4-Und-BTBT-Hex** thin films surface obtained from the solutions with a concentration of 1.0 g L^{-1} (*a*) and with a concentration of 2.0 g L^{-1} (*b*) and corresponding cross-sections along horizontal grey lines in each image (*c*, *d*) (batch 2).

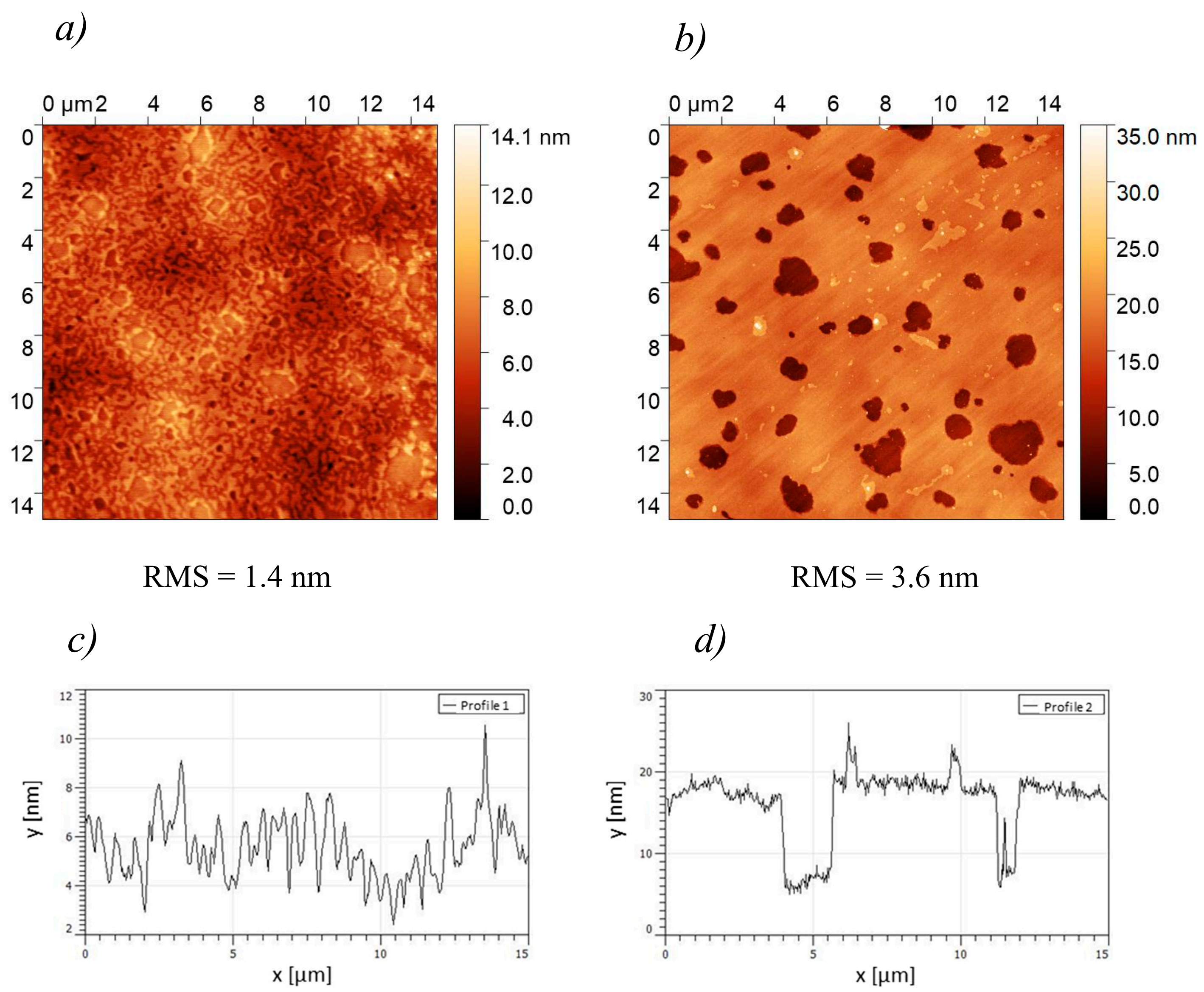

Figure S36. AFM topography images of **D4-Hex-BTBT-Oct** thin films surface obtained from the solutions with a concentration of 1.0 g L^{-1} (*a*) and with a concentration of 2.0 g L^{-1} (*b*) and corresponding cross-sections along horizontal grey lines in each image (*c*, *d*) (batch 2).

a)

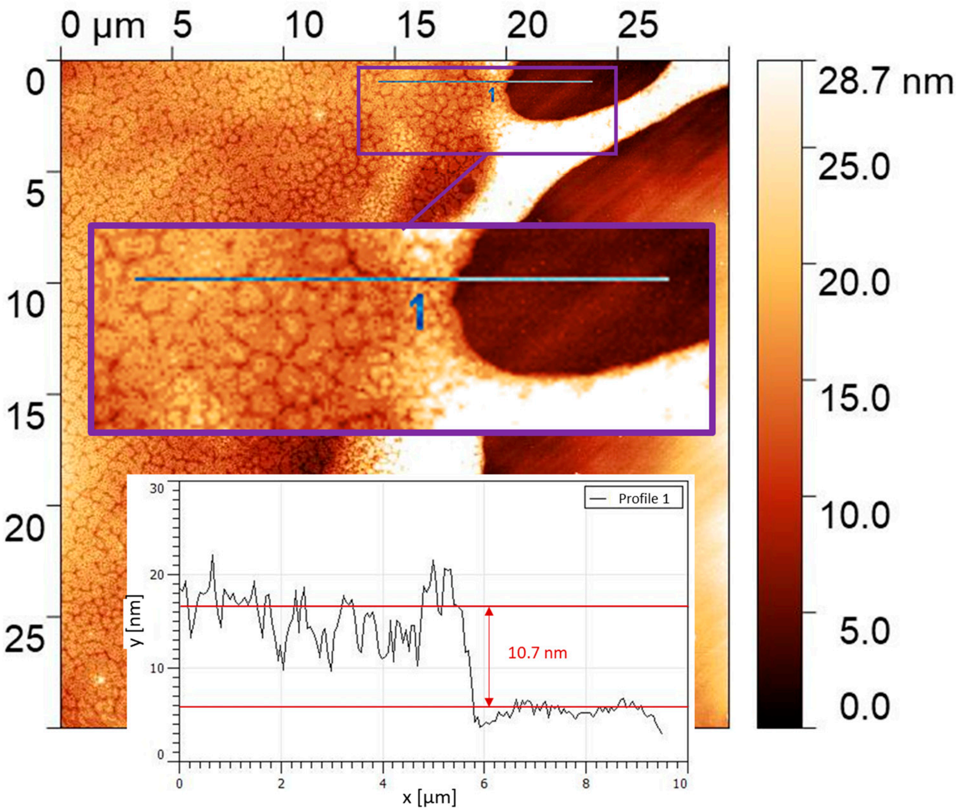

b)

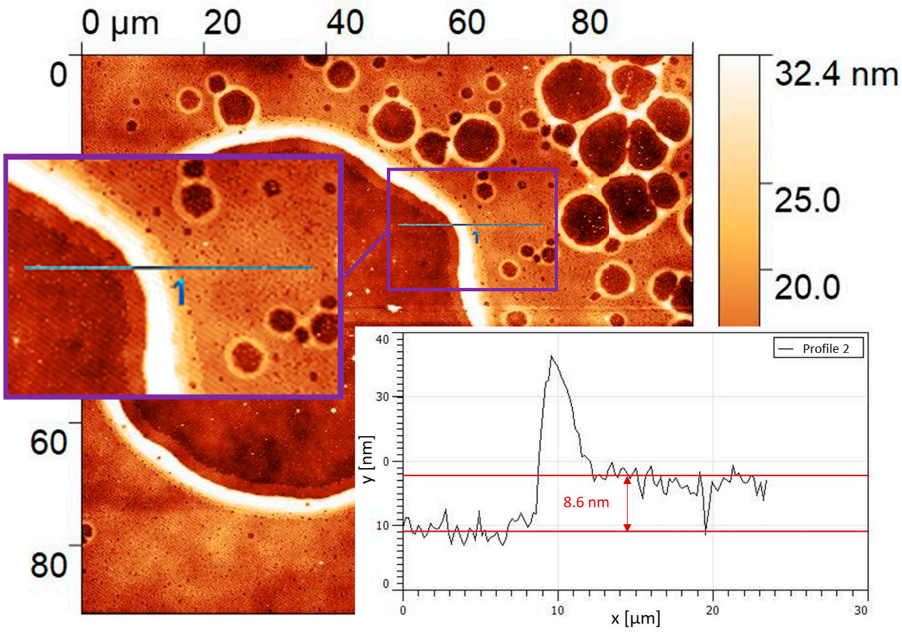

Figure S37. Thin film surface profiles of the AFM topography image fragments of **D4-Und-BTBT-Hex** (concentration of 1.0 g L^{-1}) (*a*); of **D4-Hex-BTBT-Oct** (concentration of 1.0 g L^{-1}) (*b*) (batch 2).
